# Review on the impact behavior of natural fiber epoxy based composites

**DOI:** 10.1016/j.heliyon.2024.e39116

**Published:** 2024-10-10

**Authors:** Abdu Mohammed Seid, Solomon Alemneh Adimass

**Affiliations:** Department of Mechanical Engineering, Wollo University, Kombolcha Institute of Technology, Kombolcha, Ethiopia

**Keywords:** Natural fiber, Epoxy matrix, Impact strength, Composites

## Abstract

The growing demand for sustainable and environmentally friendly materials has spurred interest in natural fiber epoxy composites as a viable substitute for synthetic-based composites. Impact strength is a crucial mechanical property for structural applications and plays a key role in successfully utilizing natural fiber epoxy composites. This comprehensive review explores the impact strength characteristics of different natural fiber epoxy composites by examining the latest research and advancements in the field. The effect of fiber content, fiber length, stacking sequence, and fiber treatment techniques, such as chemical treatments and coupling agents, on epoxy-based composites' impact strength was discussed. Additionally, the effect of fiber hybridization and formulation in determining impact strength is explored, including the curing agents, manufacturing techniques and fillers. The challenges and limitations associated with the impact strength of natural fiber epoxy composites are addressed, along with strategies to overcome them. The review concludes with a summary of the approaches to improve impact strength, including fiber selection, fiber treatment, hybridization, matrix modification, fiber orientation, curing parameters, fiber content, and fiber length. The conclusions of this review provide valuable insights into enhancing the impact strength of natural fiber epoxy composites, paving the way for their wider adoption in various industries and promoting sustainable material alternatives.

## 1 Introduction

In recent times, numerous researchers have been investigating the drawbacks associated with the use of conventional materials in structural applications. Conventional materials like steel and alloys are prone to corrosion, have less strength-to-weight ratio, possess high weight, high cost, non-biodegradable and are less resistant to naturally hazardous environments [1,2]. To address these challenges, scholars are prioritizing the creation of alternative materials like natural fiber polymer composites (NFPCs). NFPC is a widely utilized type of polymer composite that leverages the benefits of both natural fibers and polymers. These composites are created by reinforcing natural fibers with polymer as a matrix, resulting in unique properties that make them appealing for a variety of applications [1,3]. Unlike conventional synthetic materials, NFPCs are readily available, cost-effective, non-corrosive, offer high specific strength and stiffness, and are environmentally friendly, making them ideal for structural applications [4,5]. Furthermore, NFPCs have demonstrated potential in reducing material weight without compromising strength, making them well-suited for lightweight applications such as automotive components, aerospace parts, and sports equipment. The lightweight nature of NFPCs also contributes to enhanced fuel efficiency, reduced energy consumption, and improved equipment performance [4,6].

Natural fibers represent a diverse and versatile group of materials sourced from plants, animals, and minerals, with a rich history of human use spanning millennia across various applications like clothing, shelter, and everyday items. Their lower environmental footprint compared to manmade fibers has contributed to their growing popularity in a wide range of industries. Additionally, the biodegradability of natural fibers not only decreases waste and pollution but also supports local economies and traditional farming methods in different regions [4,7].

The predominant sources of natural fibers, primarily used in modern applications, are plant and animal-based. Plant-derived fibers are especially common in composite uses, with materials such as sisal, bamboo, flax, and jute being popular choices [8]. Combining natural fibers (NF) with polymer resin creates composite materials that offer distinctive mechanical properties, environmental sustainability, and suitability for engineering practices [9]. Among these properties, impact strength stands out as a critical determinant for the engineering viability of composite materials. Impact strength denotes a material's capacity to withstand sudden dynamic or shock loads without fracturing, reflecting its toughness. Therefore, evaluating a material's impact strength is crucial for determining its resistance to impacts in diverse applications like aerospace, protective gear, and automotive components [10,11]. Researchers have honed in on epoxy-based natural fiber composites, noting their ease of manufacturing, curing efficiency, high impact resistance, cost-effectiveness, and minimal shrinkage as favorable characteristics. Several studies have highlighted the considerable enhancement of impact strength achieved by incorporating natural fibers and other fillers into epoxy composites [12,13 & [[48], [49], [50], [51], [52], [53], [54], [55]].

Polymer matrix like epoxy has good impact strength due to its cross-linking structure, higher impact strength, excellent abrasion properties, and good moisture resistance [14]. Nowadays, researchers are interested in developing natural fiber epoxy-based composites to meet engineering requirements. Due to this reason, several researchers have investigated the impact strength of natural fiber epoxy-based composites. Although, a few scholars have reported a review work regarding on the natural fiber epoxy-based composite [[15], [16], [17]]. However, the current review paper focuses particularly on the comprehensive review of the impact behavior of natural fiber epoxy-based composites. The impact strength of natural fiber epoxy-based composite materials is affected by fiber content, fiber length, fiber treatment, stacking sequence, and other factors. The effects of all these factors that affect the impact strength of natural fiber epoxy-based composites are mainly discussed in this review paper. It also explores the impact strength of hybrid natural fiber epoxy composites, epoxy matrix and its properties, and a comparative study of different natural fibers on the impact performance of the epoxy-based composite. Furthermore, the conclusion and future trends are also presented.

## 2 Epoxy matrix and its properties

**Fig. 1:**
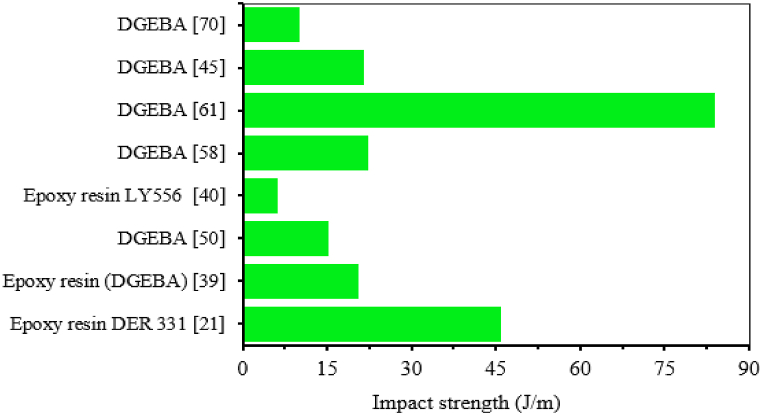
The impact strength of different epoxy matrix in terms of J/m reported by scholars.

Epoxy matrix is a versatile and commonly used thermosetting polymer that offers a myriad of applications across many industries. Compared to polyester epoxy matrix offers superior mechanical properties and plays a crucial role in construction, aerospace, electronics, coatings, and automotive applications [18,19]. Due to its better mechanical properties, epoxy resin is mostly used in polymer composite production. The major role of epoxy matrix in polymer composite is to transfer stress distribution in the fiber, to protect the fiber from moisture absorption and to produce a smooth surface finish of the composite [14]. In addition, epoxy has good compatibility and deformability with natural fibers, strong resistance to solvents and chemical attacks compared to other thermosetting matrixes. Epoxy resins undergo a curing process (often with hardeners or catalysts) that irreversibly transforms them from liquid to solid, offering excellent dimensional stability and heat resistance [16]. In general, epoxy resin retains several advantages such as excellent adhesion properties, high resistance to temperatures up to 190^0^ C, good physical and mechanical properties and low shrinkage of about 1 % compared to polyester resin. However, they have some drawbacks such as being susceptible to cracks during cured, harmful to the skin, and high cost [14]. In many research work the impact strength of pure epoxy resin revealed lower impact strength compared to natural fiber reinforced with epoxy composites. The lower impact strength epoxy resin tends to be more fragile and brittle [20]. They are subjected to chipping or cracking under a sudden impact load. This lower impact behavior of pure epoxy resins limits the use of epoxy resin in some application areas where high-impact strength is required [21,22]. Like in aerospace structural applications, the usage of epoxy is limited due to low-impact behavior, fracture toughness and delamination properties [23]. These application areas often involve vibration and sudden impacts, which can cause failure in low-impact strength of epoxy resins. To overcome the low-impact performance and limitations of epoxy resin for various application areas, researchers have employed a range of strategies to improve the impact strength of epoxy matrix [22]. The most common approach to overcome these limitations reported by the researcher is combining epoxy matrix with other natural fibers, incorporating toughening agents, and optimizing the curing process [21,24]. Many studies have focused on the role of toughening agents, apart from natural fibers and fillers, in improving the impact resistance of epoxy resin. Among the various toughening technologies available for epoxy resins are modifications of thermoplastic and thermosetting resins, liquid crystal polymers, interpenetrating polymer networks, and flexible segment curing agents. All these methods enhance the impact resistance of epoxy resin [20,25,26]. The addition of biodegradable polymer also enhances the brittleness and low impact resistance of epoxy resin. Jin et al. [27] used biodegradable polymers made from vegetable oil as toughening agents to improve epoxy resin. As can be observed, adding epoxidized soybean oil (ESO) significantly improved the impact strength of neat epoxy resin. The impact strength of neat Diglycidylether of bisphenol-A (DGEBA) exhibited a low impact strength of 13.9J/m, whereas the addition of ESO improved impact strength by 58 %. According to the authors, the addition of ESO increases the impact resistance and reduces the brittleness of the epoxy matrix because of the high intermolecular interactions between DGEBA and the ESO blends. The physical and mechanical properties of epoxy resin are shown in Table 1. The impact strength of epoxy resin without any fillers and fibers studied by many researchers reported in terms of J/m are shown in Fig. 1.

**Table 1:** Mechanical and physical properties of epoxy matrix reported by different authors.

Density (g/cm^3^)	TS (Mpa)	TM (GPa)	FS (MPa)	FM (GPa)	PE (%)	HDT (^0^C)	Viscosity at 25^0^C (kg/ms)	Ref.
1.162	110	4.1	_	_	4.6	_	_	[28]
1.16	73	5	60	_	4	100	12–13	[29,30]
_	33.86	0.712	118.73	5.781	_	_	_	[31]
_	29.3	1.66	_	_	1.1	_	_	[32]
_	57.44	0.132	48.67	0.5736	8.357	_	_	[23]
1.168	33.7	35.9	_	_	_	_	_	[33]
1.1–1.4	35–100	3–6	_	_	1–6	_	_	[33]
_	6.9	0.166	_	_	_	_	0.55	[34]
1.2–1.25	11.14	0.48	25.29	1.53	4.2–5.6	_	0.55	[35]
_	20.5	0.512	_	_	0.91	_	_	[21]
1.12–1.21	90–120	3.1–3.8	40–60	_	4	50	0.25–0.75	[36,37]

**Note:** Tensile Strength (TS), Tensile modulus (TM), Flexural Strength (FS), Flexural Modulus (FM), Heat Distorsion Temperature (HDT), Percentage Elongation (PE), Reference (Ref.).

## 3 Factors affecting the impact strength of natural fiber epoxy-based composite

### 3.1 Effect of fiber content and other filler materials

#### 3.1.1 Positive relationship with fiber content and filler material

**Fig. 2:**
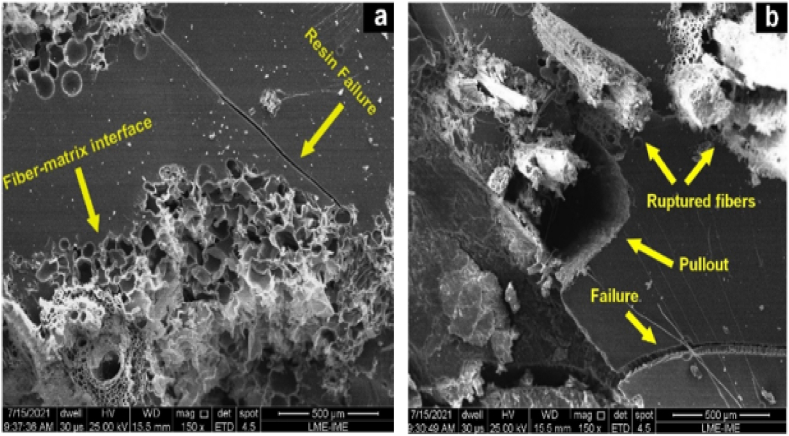
SEM images of composite (a) resin failure and (b) fiber pullouts. Reproduced with permission from Creative Commons CC BY license [39].

The content of natural fiber in the polymer matrix can change the impact strength of composite materials. The addition of fiber in a polymer matrix improves the overall properties of the matrix and reduces the cost of composites [36]. However, in some cases, the impact strength of natural fiber epoxy-based composites starts to decrease beyond an optimal fiber content due to insufficient matrix or formation of clusters, less fiber/matrix bonding and other conditions. Araya Abera et al. [13] evaluated the effect of fiber content (15–40 %) on the impact strength of randomly oriented sisal fiber-reinforced epoxy composite. The notched impact (NI) strength of the formulated composite increased with increasing fiber content up to 40 %. It can be observed that the addition of fiber significantly improved the impact strength of pure epoxy materials. Wilson N. Saba et al. [21] studied the effect of fiber content (40–60 %) on the impact strength of data palm fiber (DPF) reinforced epoxy composite. The impact strength of the composite increases by up to 50 % fiber content and slightly decreases, increasing the fiber loading by 60 %. The Authors explained that adding DPF fiber beyond 50 % reduces the impact strength due to the less wettability of fiber, leading to poor fiber/matrix bonding. Another work [38] evaluated the effect of fiber content (10–30 %) on the Izod impact strength of mallow fiber-reinforced epoxy composite. The impact strength of the composite increased along with fiber content and maximum at 30 % fiber content. Andressa Teixeira et al. [39] investigated the impact strength of caranan fiber-reinforced epoxy composite. The effect of fiber content (10–30 %) on the impact strength of the composite was analyzed. It is observed that the Izod NI strength of the composite increased with increasing fiber content and reached a maximum value of 30 % fiber content. Moreover, the SEM analysis of caranan fiber-reinforced epoxy composite at 30 % fiber loading after the impact test is shown in Fig. 2. As observed in Fig. 2a, resin failure (cracks) are created in the epoxy matrix and the formation of these crack paths is interrupted by the caranan fiber. Also, good fiber/matrix connections are shown in the SEM images at the fiber-matrix interface with increasing fiber content. On the other hand, fiber fracture and pullouts are observed in Fig. 2b. The pullout looks like a circular hole due to high energy absorption at high fiber loadings.

**Fig. 3:**
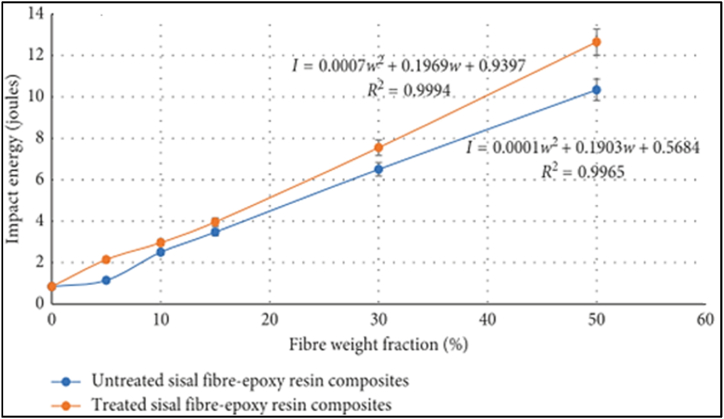
Effect of fiber weight fraction on the impact energy treated and untreated sisal fiber epoxy composite. Reproduced with permission from Creative Commons CC BY license [59].

Mishra et al. [40] studied the effect of fiber content (12–48 %) on the impact strength of bidirectional jute fiber-reinforced epoxy composite. The impact strength of the composite increased exponentially with increasing fiber content. The authors explained that the increased energy absorption of composite is due to the coupling between fiber bundles, good adhesion between the fiber and matrix, and the larger contact area between the fiber and matrix facilitated by proper fiber impregnation in the resin. Dhanalakshmi et al. [41] investigated the effect of fiber content (40–70 %) on the impact strength of areca fiber-reinforced epoxy composite. the result showed that the impact strength of the composite increased with an increase in fiber loading up to 60 %. However, the impact strength declines when the fiber content increases from 60 to 70 % fiber loadings. Srinivasa and Bharath [42] also analyzed the impact properties of treated and untreated areca fiber content (50 & 60 %) reinforced epoxy composite at different curing times. Both Izod and Charpy impact strength of 60 % treated areca fiber is higher than that of 50 % treated areca fiber for all curing times. On the other hand, processing time significantly changes the impact strength of the composite. The composite impact strength increases with increasing curing time and maximum at 1080 h. Moreover, the Charpy impact strength of the composite is higher than the Izod impact strength for all percentages of treated and untreated areca fiber-reinforced epoxy composite. Gabriel Oliveira et al. [43] studied the effect of fiber volume fraction (0–30 %) on the impact strength of the giant bamboo-reinforced epoxy composite. The Charpy NI strength of the composite increased exponentially with increasing fiber volume fraction and maximum at 30 % fiber loading. Similarly, M.K. Hussain et al. [44] also evaluated the effect of fiber content (10, 20 and 30 %) on the impact strength of bamboo/epoxy composite. The impact strength of the composite increased linearly with increasing bamboo fiber loadings. Tuan et al. [45] examined the effect of fiber content (10–25 %) on the impact strength of short banana fiber epoxy composite using compression molding techniques. The result showed an increase in impact strength when the fiber loading increases and decreases when fiber loading exceeds more than 20 % fiber loadings. Rajesh Kumar et al. [46] analyzed the effect of fiber content (0–50 %) on compression molding Phoenix species fiber-reinforced epoxy composite. The effect of fiber content on the impact strength of different sizes of Phoenix sp. fiber (300 μm, 10 mm, 20 mm, and 30 mm fibers) was considered under study. It is worth noting that fiber content increased from 0 to 40 % similarly, the impact strength also increased and maximum at 40 % fiber loading for all sizes of fiber. Whereas, the impact strength slightly deviates at 50 % fiber loading compared to 30 and 40 % fiber content. The author concluded that the addition of Phoenix sp. fiber increased the impact strength up to 258 % compared to net epoxy resin. Therefore, Phoenix species fiber plays a vital role in enhancing the impact strength of net epoxy material. Chizoba et al. [47] investigated the effect of fiber content (10–50 %) on the impact strength of a 30 mm short coir fiber-reinforced epoxy composite. Charpy NI strength of the composite increased with an increase in fiber loading up to 30 %. After optimum fiber content, the NI strength of the composite decreased with a further increase in the fiber content and minimum at 50 % fiber loading. The author remarked that the drop in NI strength after 30 % is due to insufficient fiber wetting and poor interfacial bonding between fiber and matrix. Similarly, Mohit Mittal et al. [48] also studied the effect of coir fiber content on the impact strength of coir/epoxy composite. It is worth nothing that, the impact strength of composite increased with an increase in fiber loadings. Rajesh Egala et al. [49] studied the effect of fiber content (10–40 %) on the impact strength of cortex fiber/epoxy composite. The impact strength of the composite increased almost linearly with increasing fiber loading from 10 to 40 %. The impact strength of deleb palm and carnauba fiber reinforced epoxy composite also maximum at 40 % of fiber loadings reported by Ref. [50]. Reis et al. [51] also explored the impact resistance of Amazon guaruman fiber-reinforced epoxy composite. Similarly, the Izod NI strength of the composite increased with increasing fiber content from 0 to 30 % and maximum at 30 % fiber loading. Amazon guaruman fiber significantly improved the NI strength h of net epoxy resin. Due to the brittleness nature of the epoxy matrix, composites reinforced with the lowest fiber content revealed the minimum NI strength as explained by the authors. Kang Yang et al. [11] studied the impact strength of silk fiber/epoxy composite. The impact strength of the silk fiber epoxy composite increases with increasing silk fiber content and maximum up to 60 %. Mazharuddin et al. [52] explained the effect of fiber content (0–20 %) on the impact strength of both Rose Madder and Burmese Silk Orchid fiber-reinforced epoxy composite made by hand layup technique. It was displayed that the Izod NI strength of both Rose Madder and Burmese Silk Orchid fiber-reinforced epoxy composite increased sharply with increasing fiber content and maximum at 20 g of fiber weight.H. Alamri et al. [53] studied the effect of fiber content (19, 28, 40 and 46 %) on the impact strength of recycled cellulose fiber reinforced epoxy composite. The impact strength of the composite increased sharply with increasing recycled cellulose fiber loadings. Chinapalanichamy et al. [54] investigated the effect of fiber content (25–35 %) on compression molding short, macro particle, and microparticle banana fiber-reinforced epoxy composite. The Izod NI strength of both short and macro-particle banana fiber-reinforced epoxy composite showed a sharp increase in result with increasing fiber loading. However, the NI strength of microparticle banana fiber-reinforced epoxy composite decreased with increasing fiber content. Bhuvaneshwaran et al. [55] evaluated the effect of fiber content (25–40 %) on the impact strength of different Coccinia indica fibers (CIF) length-reinforced epoxy composite. It can be seen that the impact strength of the composite increased with increasing the fiber content up to 35 %. Beyond 35 % fiber content, the impact strength of the composite is reduced. Suherman et al. [56] characterized the effect of fiber content (10–30 %) on the impact strength of kenaf fiber-reinforced epoxy composite. The impact strength of the composite increased with increasing fiber content and maximum at 30 % fiber loadings. Similarly, the impact strength of cyperus malaccensis sedge fiber reinforced epoxy composite also maximum at 30 % fiber loadings as reported by Neuba et al. [57]. Vishnuvardhan et al. [58] studied the effect of fiber content (15–25 %) on the impact strength of sisal fiber-reinforced epoxy composite. The Charpy NI strength of the composite increased linearly with increasing the fiber content up to 25 % fiber loadings. Gupta et al. [31] also analyzed the effect of fiber content (15–30 %) on the impact strength of sisal fiber-reinforced epoxy composite using the hand lay-up method. The impact strength of the composite increased linearly with increasing the fiber content up to 30 %. Similarly, Webo et al. [59] also studied the effect of fiber content (5–50 %) on the impact strength of sisal fiber-reinforced epoxy composite. The result showed that the impact strength of both treated and untreated sisal fiber-reinforced epoxy composite increases almost linearly with increasing sisal fiber content up to 50 % as shown in Fig. 3. In addition, due to the high interfacial bonding between fiber and matrix, treated fiber epoxy composite revealed better impact strength than untreated fiber epoxy composite.

**Fig. 4:**
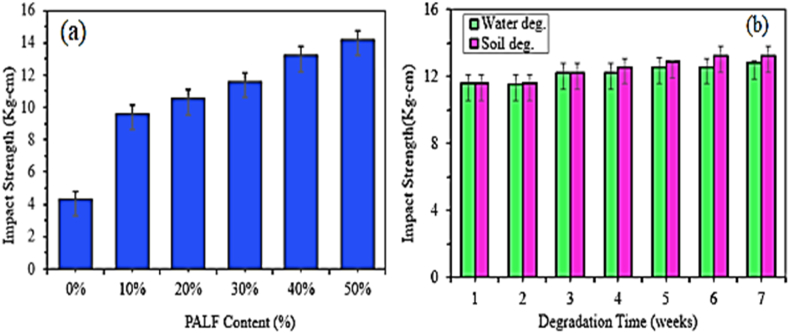
Effect of fiber content (a) and degradation time (b) on the impact strength of PALF fiber/epoxy composite. Reproduced with permission from Creative Commons CC BY license [77].

In another study, Saad et al. [60] investigated the impact strength of kenaf fiber-reinforced epoxy composite. The impact strength of the composite increased with increasing fiber content from 24.35 to 40 % and maximum at 40 % fiber loadings. Oliveira et al. [61] conducted the effect of fiber content (15–50 %) on the impact strength of fique fabric-reinforced epoxy composite. the result showed that both Charpy and Izod impact strength of the composite increased with increasing the fabric content up to 40 % and declined further with increased fique fabric content up to 50 %. In addition, the Charpy impact test revealed higher impact strength than the Izod impact test for all fiber loading. In a later study, Oliveira et al. [62] investigated the Izod impact strength of 20 % and 40 % tucum fiber-reinforced epoxy composite. It can be observed that 40 % tucum fiber reinforced epoxy composite showed higher impact strength than 20 % fiber loadings. Alhassan et al. [63] studied the effect of fiber content (30–40 %) on the impact strength of woven kenaf fiber-reinforced epoxy composite. It is observed that the impact strength of the composite increased with increasing fiber content up to 40 %. In another study, the impact strength of the Copernicia prunifera leaf fiber-reinforced epoxy composite also increased with increasing fiber content from 10 to 40 % and maximum at 40 % fiber weight [32]. Kumar et al. [64] also explored the effect of fiber content (20–50 %) on the impact strength of kenaf fiber-reinforced epoxy composite. The impact strength of the composite increased with increasing up to 40 % and decreased at 50 % of fiber content. The authors explained that beyond optimum fiber content, the shear fracture mode of failure of the composite changed to interlayer and delamination failure this is due to the less space between fiber layers, thus leading to a drop in impact strength. In another study, the impact strength of jute-reinforced epoxy composite also increased with increasing fiber content from 15 to 35 % and decreased at 51 % of fiber content [65]. Arunkumar et al. [66] characterized the impact strength of Ziziphus oenoplia Fiber-reinforced epoxy composite using the hand lay-up method. It can be observed that the impact strength of the composite increased with increasing fiber content from (30–50 %) and maximum impact strength is obtained at 50 % fiber weight fraction. Atiqah et al. [67] explored the impact strength of honeycomb natural fiber-reinforced epoxy composite. It was observed that honeycomb natural fiber content increased from 3 to 6% the impact strength decreased until it reached a maximum of 9 % filler content compared to net epoxy. The author declared that at 9 % of honeycomb natural fiber content, the epoxy matrix well bonded the filler content thus, increasing the impact strength of the composite. The lowest NI strength was obtained at 12 % filler content due to the formation of micro void and poor interfacial bonding between matrix and fiber. Lemi Demissie [68] studied the effect of false banana fiber content (20–60 %) reinforced with epoxy composite manufactured by hand layup techniques. The Charpy NI strength of the composite increased with increasing fiber content from 20 to 30 % and reaching a maximum of 30 % compared to other fiber content. After 30 % fiber content, the NI strength of the composite decreased sharply and minimum at 60 % fiber loading. R.Panneerdhass et al. [69] studied the effect of fiber content (10–50 %) on the impact strength of luffa fiber and ground nut hybrid epoxy composite. the impact strength of the composite increases with increasing fiber content up to 30 % and reduces further increase fiber content up to 50 % fiber loadings. Pereira et al. [70] evaluated the effect of fiber content (10–30 %) on the impact strength of jute fiber-reinforced epoxy composite. The impact strength of the composite increased exponentially with increasing fiber content and maximum at 30 % fiber loadings. Mathubala et al. [71] examined the effect of fiber loadings (20–35 %) on the impact strength of jute fiber-reinforced epoxy composite. It can be observed that both treated and untreated jute fiber composite increase with increasing the fiber content up to 30 % and decrease further increase the fiber content from 30 to 35 % weight. In another study, P. Kaushik et al. [72] also studied the effect of fiber content (11–55 %) on the impact strength of jute fiber reinforced with epoxy composite. It can be shown that the impact strength of the composite increased with increasing fiber content from 11 to 44 % and declined when the fiber content further increased from 44 to 55 %. In another study, Siddhant et al. [73] conducted the impact strength of short and long PLF-reinforced epoxy composite. The impact strength of both short and long PLF composite increased with an increase in the fiber content from 5 to 25 % and exhibited a maximum result at 25 % fiber loadings. Chikwendu et al. [74] studied the effect of fiber content (2–10 %) on the impact strength of coir fiber-reinforced epoxy composite by hand lay-up techniques. The Charpy impact strength of the composite increased with increasing fiber content up to 8 % and decreased with further increase in the fiber content. Kumar et al. [75] also explored the effect of fiber loadings (3–10 %) on the impact strength of coir fiber epoxy composite. again, the impact strength of the composite increased with increasing fiber content up to 7.5 %. However, the impact strength decreased with increased fiber content above 7.5 % fiber loading due to poor bonding between fiber and matrix. Similarly in another study, the impact strength of coir fiber epoxy composite increased with increasing fiber content from 10 to 30 % and decreased further increased the fiber loading beyond 30 % as reported by Sudarisman et al. [76]. The author concluded that the decrement of impact strength beyond a certain fiber content is due to less structural integrity between fiber and matrix at higher fiber loadings. Hossain et al. [77] characterized the effect of fiber content (10–30 %) on the impact strength of pineapple leaf fiber (PLF) reinforced epoxy composite. The result showed that impact strength of the composite increased almost linearly with increasing fiber content as shown in Fig. 4 (a). In addition, the impact strength of the PLF composite increased with increasing degradation time for both water and soil degradation as shown in Fig. 4 (b). From Fig. 4 (b), it can be concluded that the PLF/epoxy composite exhibited higher impact strength with water degradation than soil degradation for a 6-week duration.

Velusamy et al. [78] evaluated the influence of varying fiber content (5–30 %) on the impact strength of calotropis fiber epoxy composites with different fiber lengths. The impact strength of the composite increased with an increase in fiber content of up to 25 % fiber loadings for all fiber lengths and decreased further increase fiber loadings. Bakar et al. [79] investigated the effect of fiber content (5–25 %) on the impact strength of both treated and untreated kenaf fiber reinforced with epoxy composite. Results showed that the impact strength of both treated and untreated kenaf fiber increased with increasing fiber content up to 15 % and decline further increased the fiber content. Similarly, the impact strength of bamboo fiber-reinforced epoxy composite also increased with increasing fiber content up to 15 % and reduced at 20 % fiber loadings reported by Haixia Hu et al. [23]. The authors explained that adding bamboo fiber beyond 15 % reduced the elasticity properties of the epoxy matrix; thus, reducing the impact strength of the composite material. Manivel et al. [80] also evaluated the impact strength of gongura or kenaf fiber-reinforced epoxy composite. The impact strength of the composite increased with increasing fiber loadings from (10–15 %). However, the impact strength decreased further increasing fiber content at 20 %. Similarly, the impact strength of bagasse fiber epoxy composite increases with increasing fiber content up to 15 % and reduces the impact strength at 20 % fiber loadings reported by Nyior et al. [81]. Later, A. Balaji et al. [82] studied the effect of fiber content (5–20 %) on the impact strength of 10 mm and 20 mm banana fiber-reinforced epoxy composite. The impact strength of both 10 mm and 20 mm banana fiber reinforced epoxy composite increased with increasing fiber loading and attained maximum result at 15 % fiber loadings. However, the impact strength was reduced further increasing the fiber loading at 20 %.

**Fig. 5:**
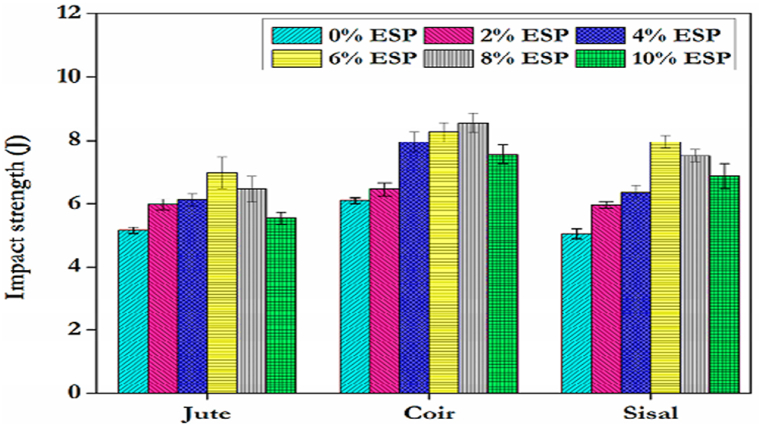
Impact strength of different fiber types with filler weight. Reproduced with permission from Creative Commons CC BY license [88].

In addition to fiber content, some filler materials significantly improved the impact strength of natural fiber epoxy-based composite. Balcioglu et al. [83] investigated the effect of silicon carbide filler content (0, 3,6, and 9 %) on the impact strength of jute/epoxy composite. The Izod NI strength of jute/epoxy composite increased with increasing silicon carbide content up to 6 % and declined after the 6 % filled percentage. The NI strength of silicon carbide-filled jute/epoxy composite increased by 15 % compared to unfilled jute/epoxy composite. Sathishkumar et al. [84] analyzed the effect of various portions of calcium carbide filler content (0, 3 % & 6 %) on the impact strength of jute/epoxy composite. The addition of 3 % and 6 % calcium carbonate filler improved the impact strength almost by 27.86 % and 42.86 % respectively compared to pure jute/epoxy composite. In a later study, Sathishkumar et al. [85] studied the impact strength of jute mat/epoxy with the addition of zinc oxide filler content of 5–25 %. The impact strength of the composite increased with increasing Zinc oxide filler content by up to 20 % and declined further increase filler content. The authors explained that the addition of zinc oxide filler improved the bonding between fiber and epoxy matrix. Lakshumu et al. [86] evaluated the addition of groundnut shell ashe fillers (3 %, 5 % and 7.5 %) effect on the impact strength of a constant 15 % banana fiber/epoxy composite. the addition of filler significantly improved the impact strength of banana fiber epoxy composites and composites of 7.5 % filler achieved higher impact strength. Santhosh et al. [87] explored the effect of fly ash content (3 %, 6 % and 9 %) on the impact strength of different concentrations of pineapple leaf fiber reinforced with epoxy composite. The addition of filler content significantly improved the impact strength of the composite. The maximum impact strength of the composite was obtained at 9 % of fly ash content and 20 % pineapple leaf fiber. however, the impact strength slightly decreases at 30 % pineapple leaf fiber with 9 % of fly ash content reinforced epoxy composite. This is due to the complex interplay of fiber dispersion, poor interfacial adhesion and fiber orientation. Avanachari et al. [88] explored the impact strength of coir, sisal and jute-reinforced epoxy composite with the addition of eggshell powder as fillers (2 %, 4 %, 6 %, 8 %, and 10 %). The impact strength of all fiber-reinforced epoxy composite increases with increasing filler weight. Coir and jute exhibited a maximum impact strength at 6 % of filler content, whereas the impact strength of sisal/epoxy composite is maximum at 8 % of filler weight. Compared to all fiber types, coir fiber/epoxy composite with the addition of eggshell powder filler revealed higher impact strength than sisal and coir/epoxy composite as shown in Fig. 5.

#### 3.1.2 Negative relationship with fiber content and filler material

Few studies have reported the negative correlation between natural fiber and the impact strength of epoxy-based composites. The impact strength of pine fiber reinforced epoxy composite decreased linearly with increased pine fiber content from 10 to 40 % weight fiber loadings as reported by Hossain et al. [89]. Similarly, M.R. Ishak et al. [90] reported the impact strength of sugar palm fiber reinforced with epoxy composite. The impact strength of the composite decreased with increasing the fiber content from 20 to 30 % of fiber loadings. In another study, the impact strength of palm seed fiber-reinforced epoxy composite also decreased with increasing fiber loadings [91].

Similar to fiber content, the impact strength of NFECs has a negative correlation with adding filler material. A paper reported by Subhendu et al. [65] confirmed the impact strength decrease with increasing cenosphere filler content on jute/epoxy composite. The authors explained that adding cenosphere filler reduces the crystallinity, thus reducing the impact strength of the composite. S.Kumar et al. [92] investigated the effect of rice husk filler (0 %, 2 %, 4 % and 6 %) on the impact strength of hybrid Bauhinia-vahlii/epoxy and hybrid Bauhinia-vahlii/sisal fiber reinforced epoxy composite. It can be observed that the addition of rice husk filler reduces the impact strength of the hybrid composite. The authors explained that due to the addition of filler, the deformability properties of the epoxy matrix were restricted.

#### 3.1.3 Discussion of fiber content and other filler material effect on the impact strength of epoxy-based composite

Many researchers have reported the effect of fiber content on the impact strength of different natural fiber-reinforced epoxy composites. The summary of the impact strength of NF epoxy-based composite with fiber content presented in this paper is illustrated in Table 2. Due to the nature of fiber type, different manufacturing techniques, test conditions, fiber matrix interactions and other conditions concluding the impact strength based on fiber content is certainly challenging. However, even if their testing method, manufacturing technique, fiber type, and other conditions are different the impact strength of natural-based composite increased with increasing fiber content up to a certain optimum fiber loadings. This increase in impact strength of natural fiber epoxy-based composite with increasing fiber content has been reported by several authors [[48], [49], [50], [51], [52], [53], [54], [55]]. As explained by the authors, the increase of impact strength with an increase in fiber content is mainly due to the good coupling between fiber bundles, good adhesion between the fiber and matrix, less fiber fraction, and the larger contact area between the fiber and matrix facilitated by proper fiber impregnation in the resin [41,42]. According to some authors, increasing fiber content in the matrix stops or blocks the crack path throughout the matrix. This helps to increase the impact energy of the composite [51]. In addition, due to the brittleness nature of the epoxy matrix by itself, the composite's energy absorption behavior is reduced when the weight of the epoxy matrix increases. On the other hand, a decrease in impact strength with increasing fiber content beyond optimum fiber content was observed similarly in many studies [[71], [72], [73], [74], [75], [76], [77], [78], [79], [80]]. This decrease of impact strength beyond optimum fiber content is due to poor bonding between fiber and matrix, due to the formation of micro void, insufficient fiber wettings and less structural integrity between fiber and matrix leads to a drop of impact strength. Moreover, due to the less space between fiber layers, the shear fracture mode of failure of the composite changed to delamination failure, thus leading to a drop in impact strength beyond optimum fiber as reported by Refs. [[75], [76], [77], [78]]. However, the impact strength of natural fiber epoxy-based composite decreases linearly with increased fiber loadings as reported by Refs. [58,59]. The main reason for the decrease in the impact strength of natural fiber epoxy-based composite with an increase in fiber loading is due to poor interfacial adhesion between fiber and matrix, fiber agglomeration, and other factors [59].

**Table 2:** Effect of fiber content on the impact strength of NFRE-based composites reported by a different author.

Fiber/epoxy composite	Fiber orientation	FT	ITM	FC (%)	OFC (%)	IS (J/m)	IS (kJ/m^2^)	Ref.
Sisal/epoxy	Random	*HL*	*Charpy, NI*	15–40	40	_	24.49	[13]
DPF/epoxy	Particle	*>>*	*>>*	40–60	50	98.71	_	[21]
Sisal/epoxy	Un-directional	*HL*	*>>*	15–30	20	_	22.03	[31]
Sedge/epoxy	150 mm long	*>>*	*Izod, NI*	10–30	30	63	_	[57]
Tucum/epoxy	1m long	*CM*	*>>*	20&40	40	75.5	_	[62]
Caranan/epoxy	≫	*HL*	*Charpy, NI*	0–30	30	151.81	_	[39]
Mallow/epoxy	Continuous	*CM*	*Izod, NI*	10–30	30	498.86	_	[38]
G.bamboo/epoxy	≫	*>>*	*>>*	0–30	30	72	_	[43]
Bamboo/epoxy	200 mm long	*HL*	*Charpy, NI*	10–30	30	_	68.88	[44]
P. Species/epoxy	10 mm	*CM*	*Izod, UI*	0–50	40	_	9.28	[46]
≫	20 mm	*>>*	*>>*	≫	50	_	9.54	≫
≫	30 mm	*>>*	*>>*	≫	40	_	9.44	≫
Caster oil cortex/epoxy	Continuous	*HL*	*Charpy, NI*	10–40	40	87.99	6.99	[49]
A.Guaruman/epoxy	150 mm long	*>>*	*>>*	0–30	30	477	_	[51]
Banana/epoxy	Random	*CM*	*>>*	10–25	20	_	12.71	[45]
≫	10 mm long	*>>*	*Charpy, NI*	5–20	15	_	2700	[82]
≫	20 mm long	*>>*	*>>*	≫	≫	_	2600	≫
Copernicia/epoxy	150 mm long	HL	*Izod, UI*	10–40	40	201.9	_	[32]
Rose madder/epoxy	200 mm long	≫	≫	5–20	20	588	_	[52]
Burmese silk/epoxy	≫	≫	≫	≫	20	543.33	_	≫
Coir/epoxy	Random	*HL*	*Charpy, NI*	10–50	30	_	26.43	[47]
≫	≫	*>>*	≫	2–10	8	100	_	[74]
≫	≫	*TM*	*Izod, NI*	3–10	7.5	_	15.47	[75]
≫	100 mm long	*CM*	*Izod, UN*	10–40	30	_	75	[76]
Jute/epoxy	Continuous	*HL*	*Charpy, NI*	15–51	35	_	10.53	[65]
≫	≫	*>>*	*>>*	10–30	30	214	_	[70]

Fiber/epoxy composite	Type of orientation	FT	ITM	FC (%)	OFC (%)	IS (J/m)	IS (kJ/m^2^)	Ref.
Coir/epoxy	20 mm short	*>>*	*Izod*	17–43	43	_	11.33	[48]
≫	Random	*>>*	*Izod, UN*	11–55	44	_	110.74	[72]
≫	20 mm short	*>>*	*Charpy, NI*	5–25	25	_	83.05	[73]
≫	200 mm long	*>>*	≫	≫	15	_	25.30	[73]
NaOH-treated Kenaf/epoxy	Random	*HPM*	Izod NI	5–25	15	_	5.12	[79]
Untreated kenaf/epoxy	≫	*>>*	≫	≫	15	_	4.46	≫
≫	10 mm short	*HL*	≫	10–30	30	_	180	[56]
≫	30 mm short	*>>*	≫	≫	30	_	140	≫
≫	Woven mat	*>>*	≫	30–40	40	_	7.28	[63]
≫	≫	*CM*	≫	20–50	40	_	13.26	[64]
NaOH-treated kenaf/epoxy	≫	*>>*	≫	≫	40	_	16.69	≫
KOH treated epoxy	≫	*>>*	≫	≫	40	_	15.24	≫
Untreated kenaf/epoxy	Chopped	*HL*	_	10–40	40	_	46.04	[60]
NaOH-treated kenaf/epoxy	≫	*>>*	_	≫	40	_	39.44	≫
Calotropies/epoxy	30 mm short	*CM*	*Izod, UN*	5–30	25	_	16.01	[78]
Bagasse/epoxy	20 mm short	*>>*	*Charpy, NI*	5–20	15	_	11.5	[81]
≫	≫	*HL*	≫	≫	≫	_	7	≫
SPF/epoxy	1 mm short	*OM*	≫	20–30	20	17.57	16.01	[90]
NaOH treated CIF/epoxy	10 mm short	*>>*	Charpy, NI	25–45	35	406.89	_	[55]
≫	20 mm short	*>>*	≫	≫	≫	425.53	_	≫
≫	30 mm short	*>>*	≫	≫	≫	580.09	_	≫
≫	40 mm short	*>>*	≫	≫	≫	438.61	_	≫

***Note:*** FT (Fabrication techniques), ITM (Impact Test Method), FC (Fiber Content), OFC (Optimum Fiber Content), HL (Hand Layup), CM (Compression Molding), NI (Notched Impact), UI (Un-notched Impact), Ref (Reference), UT (Untreated), AAT (Acrylic Acid Treatment), OM (Open molding), VIP (Vacuum Infusion Process.

**Fig. 6:**
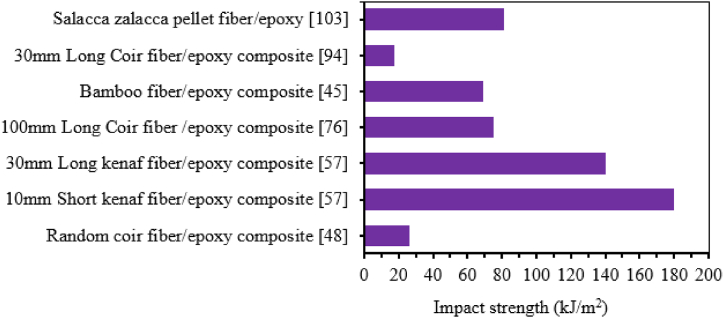
Impact strength of different natural fiber epoxy composite at 30 % fiber loading.

**Fig. 7:**
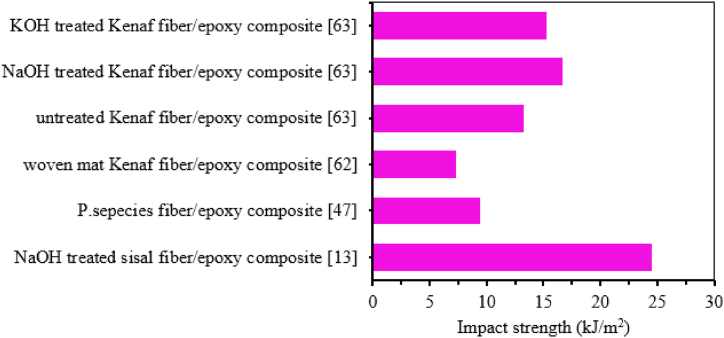
Impact strength of different natural fiber epoxy composite at 40 % fiber loading.

The impact strength of different natural fibers at optimum fiber loadings of 30 % and 40 % without considering manufacturing techniques, fiber length, testing methods, and treatment methods of fiber, covered by this paper is presented in Fig. 6, Fig. 7 respectively. It can be depicted in Fig. 6 that the impact strength of kenaf fiber epoxy-based composite exhibited maximum impact strength compared to coir, sisal, and jute fiber epoxy-based composite at 30 % fiber loadings. Similarly at 40 % of fiber loading shown in Fig. 7, KOH-treated sisal fiber/epoxy composite showed higher impact strength compared to other natural fiber/epoxy-based composites. As elaborated earlier, comparing the impact strength of natural fiber/epoxy-based composites is quite a challenge due to different use of manufacturing techniques, testing methods, treatment of fiber, and other considerations.

### 3.2 Effect of fiber length

**Fig. 8:**
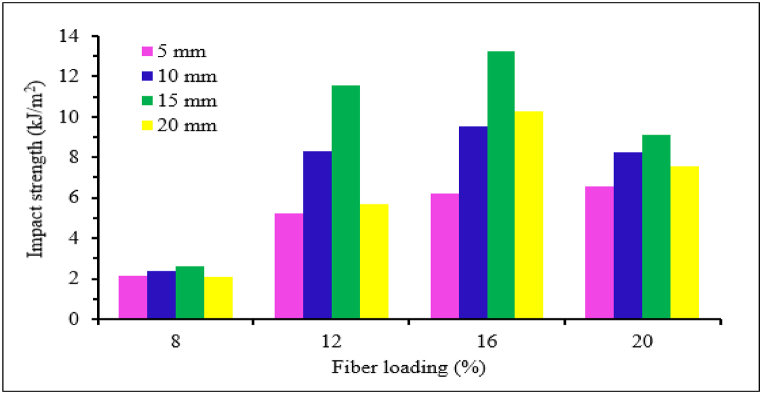
Effect of fiber length and content on the impact strength of banana fiber/epoxy composite. Adapted with permission from Elsevier, Reference [105].

The impact strength of natural fiber epoxy-based composites is a crucial parameter for any applications requiring sudden or impact load. However, the impact strength of natural fiber epoxy-based composites is often hindered by their limited impact strength. Among many other factors affecting impact strength, the fiber length variation plays a vital role in the impact strength of natural fiber epoxy-based composite. In most research works the impact strength of natural fiber epoxy-based composite increases with an increase in fiber length. This is because longer fiber transfers more load in the matrix effectively, reducing the formation of fiber pull-out during impact, reducing the crack propagation and improving the energy absorption of overall composite material. On the other hand, short fiber absorbs less impact energy and low load transfer due to less surface area in contact with the matrix. Unlike long fibers, an increase in crack propagation along the fibers is created, which leads to less energy dissipation [91,92]. Y. Leman et al. [95] studied the impact strength of short chopped and long arenga pinnata/sugar palm fiber/epoxy composite. Short chopped fiber-reinforced epoxy composite's impact strength revealed lower impact strength than long fiber-reinforced epoxy composite. The author explained that due to extensive delamination and equal dispersions of impact load into the whole lamina, the impact strength of long fiber composites resists more than that of short or chopped ones. Raghavendra et al. [96] studied the effect of treated fiber length (2, 4, and 6 mm) on the impact strength of the banana fiber-reinforced epoxy composite. The NI strength of the composite increased with increasing fiber length and maximum at 6 mm. Kongkaew et al. [97] examined the effect of fiber length (3, 5, 7, 9, and 13 mm) on hand layup fabricated 12 % vetiver fiber-reinforced epoxy composite. Similarly, the NI strength of vetiver fiber/epoxy composite increased with increasing fiber length up to 13 mm. The maximum NI strength was found at 13 mm fiber length compared to other composite as well as net epoxy material. The authors briefed that the SEM photograph of the 3 mm fiber length composite after fracture showed voids on the surface and fiber pullout, which leads to poor interfacial bonding. Jeevanantham et al. [98] also analyzed the effect of fiber length (3,5,7 and 10 mm) on the impact strength of coconut fiber-reinforced epoxy composite using hand lay-up techniques. It is observed that the impact strength of the composite increased with increasing fiber length. Maurya et al. [99] studied the effect of fiber length (5, 10, 15, and 20 mm) on the impact strength of 30 % constant sisal fiber epoxy composite. The Izod NI strength of the composite increased with increasing fiber length and maximum at a fiber length of 20 mm. Ashish et al. [100] analyzed the effect of fiber length (5,10, 15 and 20 mm) on impact strength short sisal fiber-reinforced epoxy composite fabricated by hand layup techniques. It was observed that the addition of sisal fiber significantly improved the Izod NI strength of the composite and maximum at 20 mm fiber length. Prasad et al. [93] reported the effect of fiber length (5, 10 and 15 mm) on the impact strength of different bagasse fiber-reinforced epoxy composites. It is observed that the impact strength of the composite increased with increasing fiber length. San dhyarani Biswas et al. [94] studied the effect of fiber length (5, 20 and 30 mm) on the impact strength of a 30 % coir fiber reinforced epoxy composite. The result showed that the impact strength of the composite increased with increasing fiber length and maximum at 30 mm fiber length. similarly, the impact strength of coir fiber reinforced epoxy composite showed maximum impact strength as well as the same value of impact strength at 30 mm fiber length reported by Ref. [101]. In another study, Jagadale et al. [102] also studied the effect of fiber length (5, 10 and 15 mm) on the impact strength of coir fiber epoxy composite. The impact strength of the composite increased almost linearly with increasing fiber length. Perdinan et al. [103] conducted the effect of fiber length (10, 12, 14 and 16 mm) on the impact strength of Salacca zalacca pellet fiber reinforced epoxy composite. The impact strength of the composite increased linearly with increasing fiber length and maximum at 16 mm Salacca zalacca pellet fiber. N. Venkateshwaran et al. [104] studied the effect of fiber length (5,10,15 and 20 mm) on the impact strength of different content of 8 %, 12 %, 16 % and 20 % banana-fiber reinforced epoxy composite. The result showed that the impact strength of the composite increases with an increase in both fiber length and fiber content. The maximum impact strength was obtained at 15 mm fiber length with 16 % banana fiber reinforced epoxy composite. In later study, N. Venkateshwaran et al. [105] reported the effect of fiber length (5,10,15 and 20 mm) on the impact strength of banana fiber reinforced epoxy composite. Similarly, the impact strength of the composite increased with increase in both fiber content and length and maximum at 15 mm fiber length as shown in Fig. 8. Senthilkumar et al. [106] examined the effect of fiber length (20, 30 and 40 mm) on the impact strength of a 10 % kenaf fiber reinforced epoxy composite. The impact strength of the composite increased with an increase in fiber length up to 30 mm above which the impact strength decreases at 40 mm. Rajeshkumar et al. [46] studied the effect of fiber length (300 μm particles, 10 mm, 20 mm, and 30 mm) on the impact properties of phoenix species fiber content of 0–50 % reinforced with epoxy composite. It was observed that the UI strength increased with increased fiber length up to 20 mm for 10 and 20 % of fiber loading. After 20 % fiber loading, the NI strength exhibited better results at 300 μm and 30 mm fiber length. The maximum impact strength of phoenix species reinforced epoxy composite was achieved at 300 μm with 40 % fiber loading.

**Fig. 9:**
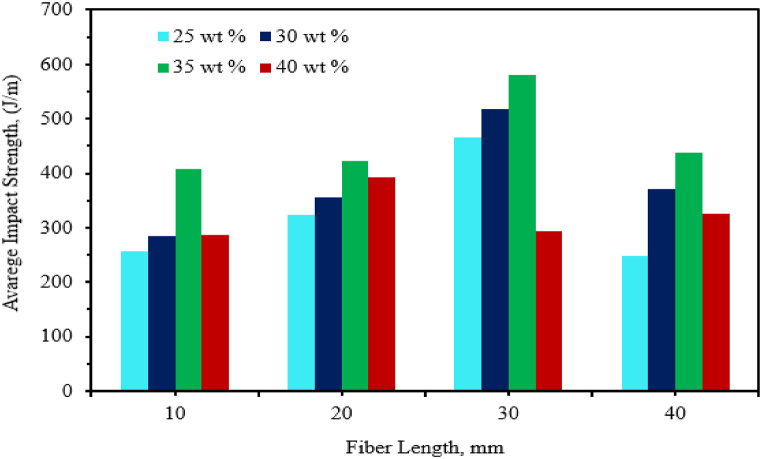
Effect of CIF content and length on the impact strength of CIF reinforced with epoxy composite. Adapted with permission from Creative Commons CC BY license [55].

Ayodeji et al. [50] studied the effect of fiber length (1 mm, 3 mm and 5 mm) and fiber content (30–40 %) on the impact strength of deleb palm fiber reinforced epoxy composite. The impact strength of the composite increased with increasing fiber length for all fiber loadings. Bhuvaneshwaran et al. [55] evaluated the effect of fiber length (10, 20, 30 and 40 mm) on the impact strength of CIF-reinforced epoxy composite. It can be observed that the impact strength of the composite increased with increasing fiber content up to 35 % in all fiber lengths and the highest impact strength was obtained at 30 mm fiber length. As shown in Fig. 9, an increase in fiber length and fiber content beyond optimum result reduces the impact strength of the composite. Bisaria et al. [107] investigated the effect of fiber length (5,10,15 and 20 mm) on the impact strength of randomly oriented short jute fiber-reinforced epoxy composite. The impact strength of the composite increased with increasing fiber length. Sharath et al. [108] analyzed the effect of fiber length (3 mm, 6 mm, 9 mm, 12 mm & 15 mm) on the impact strength of 50 % hybrid coir and wild date Palm fiber–reinforced epoxy composite. The impact strength of natural fiber hybrid epoxy composite increased almost linearly with increasing fiber length and maximum at 15 mm fiber length.

In most studies, the impact strength of natural fiber/epoxy composite increased with increasing fiber length discussed earlier. However, in some research work the length of fiber and impact strength has a negative relation. Suherman et al. [56] conducted the effect of fiber length variation (10 and 30 mm) on the impact strength of kenaf fiber/epoxy composite. The impact strength of 10 mm kenaf fiber length revealed a maximum result compared to 30 mm fiber length. The impact strength of the composite at 30 mm fiber length revealed lower impact strength compared to 10 mm fiber length due to the formation of agglomeration in the composite. Mylsamy and Rajendran [109] studied the impact strength of 3 mm, 7 mm and 10 mm agave fiber/epoxy composite. The impact strength of agave fiber epoxy composite decreased linearly with increasing the fiber length up to 10 mm. Sadeq et al. [110] also analyzed the effect of grain size (250, 750, and 950 μm) on the impact strength of coir fiber-reinforced epoxy composite with 10 % fiber content. The Charpy NI strength of the composite decreased with increasing the grain size. On the other hand, the addition of grain size significantly improved the NI strength of the composite compared to net epoxy material. The NI strength is maximum at a grain size of 250 μm. This is due to the reason that at small grain size, the composite has good interfacial bonding and good interaction of fiber with the matrix. Thus, helps to resist crack formation and increases the NI strength of coir fiber-reinforced epoxy composite. The effect of fiber length on the impact strength of natural fiber epoxy based composites are tabulated in Table 3.

**Table 3:** The impact strength of NF-epoxy composite with different fiber length.

Natural Fiber/epoxy composite	Fabrication techniques	Fiber length (mm)	Impact Strength (kJ/m^2^)	Impact strength (J/m)	Ref
20 % banana fiber/epoxy	Hand layup	2	_	17.6	[96]
		4	_	19.9	
		6	_	20.3	
12 % vetiver fiber/epoxy	≫	3	13.47	_	[97]
		5	13.74	_	
		7	14.40	_	
		9	18.37	_	
		13	19.49	_	
	≫	5	7.008	_	[99]
20 % sisal fiber/epoxy		10	13.66	_	
		15	18.38	_	
		20	27.63	_	
	≫	5	8.74	_	[100]
Sisal fiber/epoxy		10	15.79	_	
		15	21.86	_	
		20	31.96	_	
Cos 8436 SBF/epoxy	≫	15	_	3.7	[93]
Cos 767 SBF/epoxy	≫	5	_	3.5	
CoJ88 SBF/epoxy	≫	5	_	3.5	
CoJ0239 SBF/epoxy	≫	15	_	4.5	
		0.25	5.45	_	[110]
10 % coir fiber/epoxy	≫	0.75	6.68	_	
		0.95	8.69	_	
		5	16	_	[94]
30 % coir fiber/epoxy	≫	20	16.5	_	
		30	17.5	_	
		5	7.15	_	[102]
5 % coir fiber/epoxy	≫	10	8.20	_	
		15	9.785	_	

Natural Fiber/epoxy composite	Fabrication techniques	Fiber length (mm)	Impact Strength (kJ/m^2^)	Impact strength (J/m)	Ref
10 % kenaf fiber/epoxy		20	_	10	[106]
	Hand layup	30		70	
		40		50	
30 % salacca zalacca pellet fiber/epoxy	≫	10	50.43	_	[103]
		16	80.93	_	
Hybrid 50 % Coir and 50 % date palm fiber/epoxy		3	2.63	_	[108]
		6	3.05	_	
	≫	9	3.52	_	
		12	4.11	_	
		15	6.41	_	
30 % kenaf fiber/epoxy	Hot pressing method	10	140	_	[56]
		30	180	_	
40 % Coccinia indica fibers/epoxy	Compression molding	10	_	286.70	[55]
		20	_	391.89	
		30	_	293.45	
		40	_	325.94	
35 % Coccinia indica fibers/epoxy	≫	10	_	406.89	[55]
		20	_	423.53	
		30	_	580.09	
		40	_	438.61	
30 % Coccinia indica fibers/epoxy	≫	10	_	284.09	[55]
		20	_	355.17	
		30	_	518.53	
		40	_	371.84	
25%Coccinia indica fibers/epoxy	≫	10	_	256.80	[55]
		20	_	324.09	
		30	_	466.82	
		40	_	247.80	
16 % banana fiber/epoxy	Hand layup	5	2.62	_	[105]
		10	11.56	_	
		15	13.25	_	
		20	9.12	_	

***Note:*** SBF (Sugarcane Bagasse Fiber), Ref. (Reference).

In summary, the impact strength of long fiber reinforced epoxy composite revealed higher impact strength than short fiber/epoxy composite keeping other parameters constant. For the case of long fiber/epoxy composite, the impact strength is increased due to high energy dissipation, less fiber pulls out of the matrix and the applied impact load is distributed effectively in the composite. On the other hand, short fiber/epoxy composite exhibits less impact strength than long fiber/epoxy composite due to the formation of localized stress, fiber/epoxy breakage, fiber/epoxy debonding and fiber pullout this leads to less dissipation of energy. In addition, the lower impact strength was observed due to the formation of much longer fiber pull-out, and poor interface bonding between fiber and matrix which leads to crack propagation in the composite easily.

### 3.3 Effects of chemical and other treatments

**Fig. 10:**
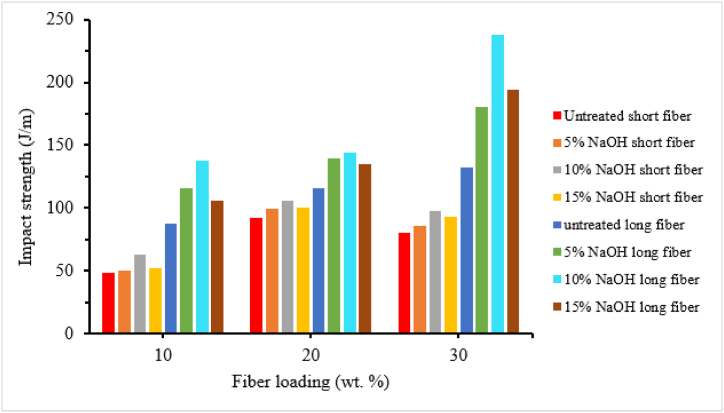
Effect of alkali treatment on the impact strength of short and long Napier grass fiber reinforced epoxy composite. Adapted from [121].

The strength of composite material relies significantly on the bonding between the fiber and matrix. Enhancing this interfacial bonding through fiber surface treatment is vital. Surface treatments aid in cleaning, preventing moisture absorption, and improving surface smoothness. These actions directly enhance the bond between the matrix and fiber, thereby boosting the mechanical strength of the composite material [111,112]. Various methods, including chemical treatments (such as alkali treatment, acylation, saline treatment, esterification, peroxide treatment, enzymatic treatment and malic anhydride propylene (MAP), etc.) and physical treatments (like plasma treatment, corona treatment, electron beam irradiation and autoclave treatment, etc.), are commonly employed to enhance the properties of natural fibers [113]. Chemical treatments are the most commonly utilized treatment to remove lignin from the fiber surface to roughen it, thereby improving fiber-matrix interaction. The impact strength of composite materials is predominantly influenced by fiber rigidity and aspect ratio. Chemical treatments reduce fiber diameter, increase surface area, aspect ratio, and enhance bonding between the fiber and matrix, ultimately improving impact strength compared to untreated fiber composites [114]. Suresh et al. [115] evaluated the impact strength of 5 % NaOH treated and untreated coconut shell fiber reinforced epoxy composite. Treated coconut shell fiber epoxy composite revealed higher impact strength compared to untreated one. The author explained that alkali treatment removes the lignin, hemicellulose and other unnecessary materials presented on the surface of fiber this leads to better interfacial bonding between fiber and matrix in the composite. On the other hand, less impact strength occurred in untreated fiber epoxy composite due to agglomeration on the fiber surface, leading to poor fiber-matrix bonding. Bachtiar et al. [116] studied the effect of 0.25 M and 0.5M NaOH solution with different soaking times of (1h, 4h and 8h) on the impact strength of sugar palm fiber reinforced epoxy composite. The impact strength of the composite increased with increased soaking time for both NaOH concentration. However, the impact strength of 0.25M NaOH treated sugar palm fiber epoxy composite have lower impact strength compared to untreated fiber epoxy composite. Whereas, 0.5 M solution gives better impact strength at 4h and 8h soaking time compared to untreated sugar palm fiber reinforced epoxy composite. Samsul Rizal et al. [117] characterized the impact strength of 5 % NaOH treated with a soaking time of (1h, 2h, 4h and 8h) and untreated typha fiber epoxy composite. The result demonstrated that alkali-treated typha fiber had a higher impact strength than untreated typha fiber reinforced epoxy composite. Furthermore, the impact strength of the composite increased with an increase in soaking time up to 2 h, but decreased significantly with a further increase in soaking time. Karthikeyan et al. [101] studied the effect of NaOH and sodium lauryl sulfate (SLS) concentration (2 %, 4 %, 6 %, 8 % and 10 %) on the impact strength of different coconut fiber lengths (10, 20 and 30 mm) reinforced epoxy composite. It can be observed that the impact strength of both NaOH and SLS treated coir fiber reinforced epoxy composite increased with increasing the amount of concentration up to 6 % and decreased further increase amount of concentration. However, SLS treatment revealed maximum impact strength compared to NaOH treatment in all fiber lengths and maximum impact strength was obtained at 30 mm fiber length. Venkateshwaran et al. [118] reported the effect of NaOH concentration (0.5 %, 1 %, 2 %, 5 %, 10 %, 15 % and 20 %) on the impact behavior of banana fiber/epoxy composite. The impact strength of 1 % NaOH-treated banana fiber/epoxy composite is higher than other concentrations. Muhammad et al. [12] studied the impact strength of 6 % NaOH treated and untreated kenaf fiber-reinforced epoxy composite. It can be observed that the impact strength of treated fiber epoxy composite is higher than untreated kenaf fiber epoxy composite. The authors explained that alkali treatment removes waxy substances and impurities and makes the fiber surface rougher, thus increasing the bonding between fiber and matrix. Sathish et al. [64] investigated the effect of NaOH and KOH concentrations (3 %, 6 % and 9 %) on the impact strength of a constant 40 % kenaf fiber/epoxy composite. The impact strength of composite sample revealed a maximum result at 6 % of NaOH and KOH out of three concentration. As the authors explained, the interlocking between kenaf fiber and epoxy matrix as well as aspect ratio was improved at this concentration. However, beyound 6 % concentration resulted in reduced impact strength and fiber damage. This was caused by excessive chemical treatment, which led to delingnification. Srinivasa and Bharath [42] also analyzed the impact strength of 10 % KOH (Potassium Hydroxide) treated areca fiber and untreated areca fiber reinforced epoxy composite. Untreated areca fiber epoxy composite exposed the lowest impact strength compared to treated areca fiber epoxy composite. The chemical treatment enhances the fiber matrix interfacial bonding due to this treated areca fiber epoxy composite achieving maximum impact strength. Santhosh et al. [119] analyzed the impact strength of treated and untreated banana fiber-reinforced epoxy composite. The fibers are kept constant with a fiber loading of 30 %. It was shown that 5 % NaOH-treated banana fiber has higher impact strength than untreated banana fiber-reinforced epoxy composite. The reason behind this is alkali treatment improves the interfacial bonding between fiber and matrix by removing lignin, hemicellulose, and other unnecessary components of the fiber. Mathubala et al. [71] examined the impact strength of 0.06 M sodium per iodate treated and untreated jute fiber epoxy composite. The result showed that treated fiber epoxy composite revealed higher impact strength than untreated fiber epoxy composite. A. Premnath [120] reported the effect of 10 % NaOH treatment on the impact strength of hybrid sisal/jute fiber reinforced epoxy composite. It can be seen that chemical treatment affected the impact strength of the composite. The impact strength of treated hybrid jute/sisal reinforced epoxy composite exhibited better impact strength compared to untreated composite. Girisha et al. [6] also studied the effect of 10 % NaOH treatment on the impact strength of areca nut husk and tamarind fruit fibers reinforced epoxy hybrid composite. It can be observed that the impact strength of treated fiber-reinforced hybrid epoxy composite is higher than that of untreated fiber-reinforced epoxy composite. This is because 10 % NaOH improved the impact strength of a composite by removing the lignin and hemicellulose part of the fiber, thus increasing the surface area and interfacial bonding of the composite. Kommula et al. [121] also studied the effect of NaOH concentration (5 %, 10 % and 15 %) on the impact strength of short and long-strand Napier grass fiber/epoxy composite. The impact strength of both short and long-strand fiber/epoxy composite increased with increasing alkali concentration up to 10 %. In addition, the impact strength of the composite increases with an increase in fiber loadings up to 20 % for all alkali concentrations as shown in Fig. 10.

**Fig. 11:**
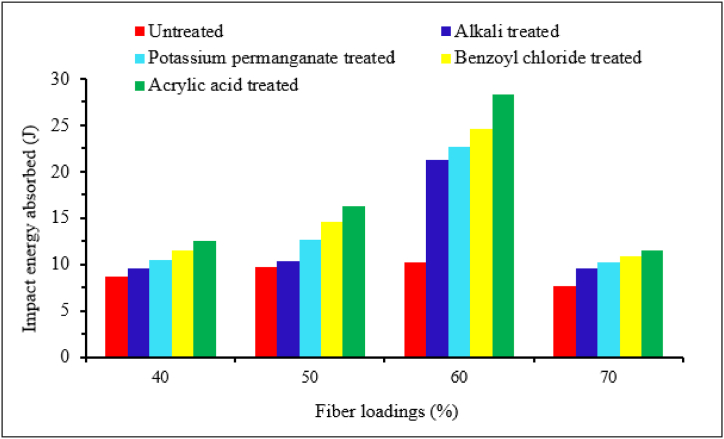
Impact energy absorbed by areca fiber reinforced epoxy composites with different chemical treatments and fiber loadings. Adapted with permission from Creative Commons CC BY license [41].

Some researchers used different chemical treatments to identify the best treatment method for natural fiber composite. Dhanalakshmi et al. [41] studied the effect of different chemical treatments (alkali treated, potassium permanganate, benzoyl chloride and acrylic acid treated) on the impact strength of areca fiber reinforced epoxy composites. It can be observed that all type of chemical treatment enhances the impact strength of the composite compared to untreated epoxy composite for all fiber loadings as shown in Fig. 11. Moreover, acrylic acid-treated areca fiber epoxy composite exhibited the highest impact strength than other chemical treatments in all fiber loadings. The authors explained that acrylic acid is very efficient for areca fiber treatment and better enhances the fiber and matrix bonding of the composite. Similarly, Ramadevi et al. [10] evaluated the impact strength of NaOH, benzene diazonium chloride, permanganate and acrylated treated abaca fiber reinforced epoxy composite. The effect of this chemical treatment on the impact strength of 10, 20, 30, 40, 50 and 60 % of abaca fiber loadings was evaluated. The result showed that benzene diazonium chloride treated abaca fiber composite revealed higher impact strength compared to other chemical treatments and maximum at 40 % fiber loadings. The authors explained that at 40 % fiber loadings, there is a good distribution of load from fiber to matrix, less fiber breakage and better interfacial bonding between fiber and matrix. In addition, the impact strength of the composite showed a similar fashion in all fiber loadings.

#### 3.3.1 Discussion of chemical treatment effect on the impact strength of epoxy-based composite

The impact strength of NFECs is influenced by matrix properties, fiber properties and matrix/fiber interface properties. Many researchers have used chemical treatments for natural fiber to change the properties of fiber that are more compatible with the matrix and to improve the interface properties by enhancing fiber-matrix bonding. Therefore, different techniques of fiber treatment, different concentrations and soaking times have been employed to improve the impact strength of NFECs. Regardless of treatment type, percentage of concentration and soaking time treated NFECs exhibited higher impact strength than untreated NFECs reported in many research works. This is because chemical and other treatment methods improve the impact strength of NFECs by removing unwanted compositions of fiber like lignin, hemicellulose and others to enhance the interfacial bonding between fiber and matrix [10,19,63], [[115], [116], [117], [118], [119], [120]]. The concentration of chemical treatment significantly affected the impact strength of the composite as reported by many authors. It can be observed that the impact strength of NFECs increases with increasing concentration of chemicals up to a certain optimum weight. However, the impact strength decreases with increasing NaOH concentration this is due to the reason that at high concentrations of NaOH treatment, the main constitute of the fiber i.e. cellulose is damaged thus reducing the impact resistance of the composite. The other factor affecting the impact strength of composite related to the chemical treatment of fiber is soaking time. Similarly, the impact strength of NFECs increases with increasing soaking time up to a certain optimum time then after that decrease in impact strength was reported. This reduction of impact strength is due to the damage to the cellular structure of the fiber during a long duration of alkali concentration [113,114]. Irrespective of concentration and soaking time, some researchers reported the impact strength of NFECs with different types of chemical treatment to differentiate the best and most efficient treatment method. It has been reported that benzene diazonium chloride treatment showed the highest impact strength followed by acrylic acid, benzoyl chloride, permanganate, and alkali (NaOH) treatment respectively in decreasing order [10,42].

### 3.4 Effect of stacking sequences

**Fig. 12:**
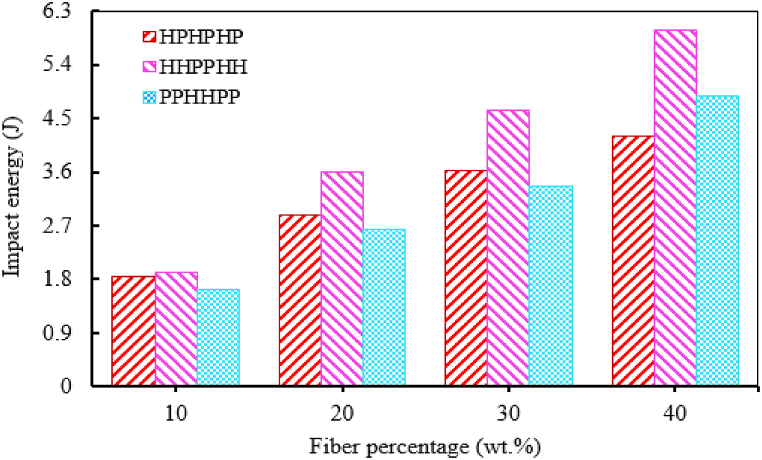
Effect of stacking sequence on the impact strength of hybrid palmyra and hemp fiber epoxy composite. Reproduced with permission from Creative Commons CC BY license [125].

One of the key factors that change the properties of natural fiber-reinforced epoxy composites is the stacking sequence of the fiber layers. Natural fiber-reinforced epoxy composites' impact behavior is affected by the stacking sequence or fiber orientation of natural fiber other than fiber content, fiber length and other parameters that affect its impact strength. The stacking sequence can affect the stress transfer mechanisms, energy dissipation, and failure modes within the composite, thereby influencing its impact resistance. The fiber orientation on the impact strength of natural fiber epoxy composite is related to the direction of fiber and applied impact load. Unidirectional fiber-oriented epoxy composites exhibited maximum impact strength when the applied impact load is perpendicular to fiber orientation [[122], [123], [124], [125]]. Rapeta Sundara et al. [122] explored the effect of stacking sequence (0, 15, 30, and 45°) on the impact strength of hemp, flax, and kenaf fiber-reinforced epoxy composite. The NI strength of all hemp, flax and kenaf fiber-reinforced epoxy composite showed a zigzag result with increasing stacking sequence. The NI strength is maximum at 0°-fiber orientation and minimum at 45°-fiber orientation for all three fiber-reinforced epoxy composites. This indicates the orientation of the fiber perpendicular to the applied load exhibits maximum results. Kumaresan et al. [124] analyzed the effect of fiber orientation (90°, ±45°^,^ and 0/90°) on the impact strength of sisal fiber epoxy composite. The NI strength of the composite is maximum at 90°-fiber orientation and minimum at cross-ply lamina. This shows that impact strength is maximum when the fiber orientation is perpendicular to the direction of applied force. Varanasi et al. [125] investigated the effect of stacking sequence for hybrid hemp (H)/Palmyra (P) reinforced epoxy composite. The Izod NI strength of different stacking sequences i.e. HPHPHP, HHPPHH and PPHHPP composite with (10, 20, 30, and 40 %) fiber content were analyzed. Hybrid HHPPHH reinforced epoxy composite exhibited the highest NI strength compared to the HPHPHP and PPHHPP stacking sequence as shown in Fig. 12.

Another study work [126] reported the effect of stacking sequence on the impact strength of hybrid Kenaf (k)/Jute (J) fiber-reinforced epoxy composite. The impact strength of KJJK composite showed a higher value than other stacking sequences i.e. JKKJ, JKJKA, and untreated JKKJ reinforced epoxy composite. P. Sathish et al. [127] analyzed the impact strength of kenaf-banana hybrid reinforced epoxy composite with different orientations. The result showed that hybrid 45°-oriented hybrid composite has higher impact strength than other orientations of banana-kenaf hybrid composite. Sathyaseelan et al. [128] also evaluated the effect of stacking sequence on the impact properties of hybrid Areca (A)/Kenaf (K) fiber-reinforced epoxy composite. The result revealed that composites having all areca fiber exhibited the highest NI strength compared to other lamina configurations. While composites having all kenaf fiber revealed the lowest NI strength. In addition, hybrid epoxy composites having an outer layer made from areca fiber and kenaf fiber with center resists more impact energy than other stacking sequences. Daniel et al. [129] studied the effect of stacking sequence on the impact strength of hybrid jute (J)/bamboo (B) fiber-reinforced epoxy composite. The impact strength of the JBJ stacking sequence exhibited the lowest result compared to JJJ, BBB, and BJB laminated composite. BJB stacking sequence showed the highest impact strength compared to another stacking sequence. Jawaid et al. [130] studied the effect of stacking sequence on the impact strength of hybrid jute/empty fruit bunch (EFB) fiber-reinforced epoxy composite. It can be observed that hybrid EFB/Jute/EFB fiber-reinforced epoxy composite revealed higher impact strength than hybrid jute/EFB/jute fiber-reinforced epoxy composite. N. Rajini et al. [131] studied the effect of angle ply on the impact strength of jute fiber woven mat/epoxy composites immersed in water for 10, 20, 30 and 40 days of aging. The result showed that the impact strength of [0°/0°/0°/0°/0°] ply laminate exhibited maximum result compared to [0°/+30°/0°/-30°/0°] angle-ply laminate and [0°/+45°/0°/-45°/0°] angle-ply laminate in all 10, 20, 30 and 40 days aging of the composite. Venkatesh et al. [123] evaluated the effect fiber orientation on the impact strength of unidirectional, bidirectional and multilayer oriented hybrid kenaf/sisal fiber reinforced epoxy composite. The unidirectional kenaf/epoxy, sisal/epoxy and hybrid kenaf/sisal epoxy composite revealed higher impact strength compared to bidirectional and multidirectional oriented composite. Similarly, unidirectional water hyacinth fiber reinforced epoxy composite showed higher impact strength than woven mat water hyacinth fiber reinforced epoxy composite [132]. Dattatray et al. [133] studied the impact strength of multilayer and parallel layer-oriented banana fiber epoxy composite. It can be observed that the impact strength of parallel layer-oriented composite revealed higher impact strength than multilayer-oriented composite.

## 4 Impact strength of hybrid natural fiber epoxy-based composite

**Fig. 13:**
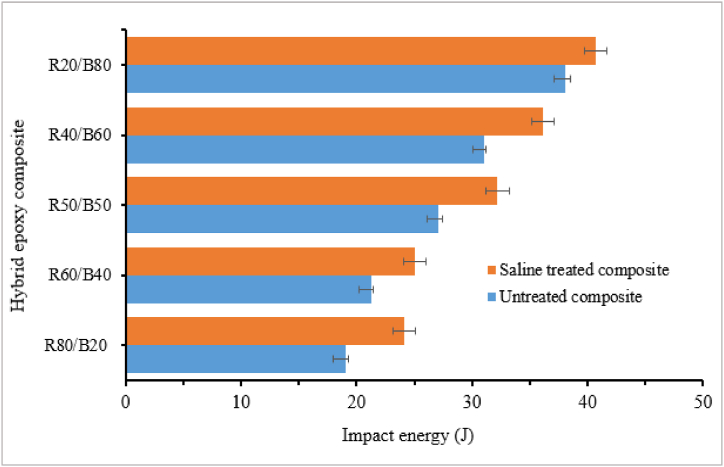
Impact strength of Ramie/Banana fiber reinforced hybrid epoxy composite. Adapted with permission from Creative Commons license [140].

Hybrid natural fiber polymer composites are formed by combining more than two different natural fibers reinforced with polymer matrixes. Researchers reported that hybridizing different natural fibers with constant or varying weight fractions gives better mechanical, physical and cost-effective composite materials than single-fiber polymer composites Several authors study the impact strength of hybrid natural fiber epoxy-based composites [134,135]. Kumar et al. [136] explored the impact strength of hybrid hemp/sisal reinforced epoxy composite manufactured by hand lay-up technique. It was observed that Charpy NI strength of hybrid fiber reinforced epoxy composite increased with increasing sisal fiber content from 0 to 100 % and dropped its result at an equal portion of hemp 50 % & sisal 50 % fiber content. The maximum NI strength of the composite was obtained at 100 % sisal & 0 % hemp-reinforced epoxy composite. Similarly, N. Venkateshwaran et al. [105] studied the impact strength of hybrid banana/sisal reinforced epoxy composite. The impact strength of hybrid composite increases almost linearly with increasing sisal fiber content from 0 to 100 % and maximum impact strength is obtained at 0 % banana/100 % sisal-reinforced epoxy composite. Mohan et al. [137] also evaluated the impact strength of hybrid sisal/bamboo reinforced epoxy composite. The composite's impact strength significantly improved by adding sisal fiber. Hybrid composites with 12.5 % and 7.5 % bamboo fiber content revealed maximum impact strength. Cavalcanti et al. [134] investigated the effect of hybridization on the impact strength of jute/epoxy composite. Hybrid jute/sisal/epoxy and jute/curaua/epoxy composite showed an impact strength of 211 % and 111 % increase compared to non-hybrid jute/epoxy composite respectively. Boopalan et al. [135] studied the impact behavior of hybrid jute/banana reinforced with epoxy composite. The addition of banana fiber increases the impact strength of the hybrid composite, and the maximum strength is obtained at 50 % jute +50 % banana fiber/epoxy composite. Atmakuri et al. [138] analyzed the impact strength of caryota (C) and sisal (S) fibers reinforced with a constant 60 % epoxy composite. The impact strength of hybrid composite increased with increasing sisal fiber content from 15 to 25 % and maximum at 15 % C/25 % S hybrid fiber composite. The impact strength of both single caryota and sisal fiber reinforced epoxy composite showed lower impact strength compared to hybrid composite. Prabhu. L et al. [139] study the impact strength of hybrid waste tea and sisal fiber reinforced epoxy composite. The impact strength of the hybrid composite increased with increasing sisal fiber content. On the other hand, an equal weight of sisal/waste tea/epoxy composite achieved maximum impact strength. Girisha et al. [6] examined the impact strength of treated and untreated areca nut husk and tamarind fruit fibers reinforced epoxy hybrid composite. Effects of fiber content (10–50 %) on the Izod impact strength of hybrid composite were analyzed. It was observed that the Izod impact strength of both treated and untreated fiber hybrid reinforced epoxy composite increased with increasing fiber content up to 50 % fiber content. Moreover, treated hybrid fiber composite revealed higher impact strength compared to untreated hybrid fiber composite. T. Raja et al. [140] also evaluated the effect of fiber content (20–80 %) on the impact strength of both saline-treated and untreated ramie/banyan fiber-reinforced epoxy composite. As shown in Fig. 13, both treated and untreated hybrid composite showed an increase in impact strength with increasing ramie fiber content. Similar to other studies treated hybrid fiber/epoxy composite revealed a 9 % improvement in impact strength compared to untreated hybrid fiber/epoxy composite for all fiber loadings. The author noted that due to its superior physical properties ramie fiber can enhance the impact strength of hybrid composites compared to banana fiber.

Lakshmi et al. [141] characterized the impact strength of hybrid bagasse (B)/rice husk (RH) fiber-reinforced epoxy composite. Different proportions of fiber (i.e. B 30 % & RH 20 %, B35 % & RH 15 %, and B 40 % & RH 10 %) reinforced with 50 % epoxy were used for characterization. It was marked that the Izod NI strength of the composite significantly improved with increasing bagasse fiber content and decreasing rice husk fiber correspondingly. The maximum NI strength of the composite was found at B 40 % & RH10 % fiber-reinforced epoxy composite. Srinivasa Rao et al. [142] explored the impact strength of hybrid narepa/uttareni fiber with and without bio filler reinforced with 30 % epoxy composite. The NI strength of hybrid narepa/uttareni fiber-reinforced epoxy composites is higher compared to narepa/uttareni fiber filled with 10 % range peel powder reinforced with epoxy composite. Mohit et al. [35] studied the impact strength of pinapple hybrid leaf/coir fiber reinforced epoxy composite. it can be noted that, the brittlness of pure epoxy resin improved significantly by the addition of both pinapple and coir fibers. On the other hand, the impact strength of the hybrid composite decreased with an increase in coir fiber content.In another study, Boopathi et al. [143] characterized the impact strength of different hybrid natural fibers i.e. jute, coir, cotton, flax, sisal, human hair, calotropis, and kenaf with constant 20 % coir fiber loadings reinforced with 64 % of the epoxy composite. The result showed that the impact strength of the coir fiber-reinforced epoxy composite is 2.4 kJ/m^2^, which is higher than the impact strength of hybrid coir/human hair, coir/calotropis, and coir/cotton fiber reinforced epoxy composite. Whereas, hybrid coir/jute, coir/flax, coir/sisal, and coir/kenaf fiber reinforced epoxy composite showed an increase of 13.3 %, 15.4 %, 10.4 %, and 18.8 % of impact strength compared to single coir fiber/epoxy composite respectively. The impact strength of hybrid coir/kenaf fiber reinforced epoxy composite exhibited higher result compared to other hybrid composites. This is due to the good interaction of kenaf fiber with coir and good interfacial bonding between matrix and fiber. In another study, Varanasi et al. [125] investigated the effect of fiber content (10, 20, 30, and 40 %) on the impact strength of hybrid hemp/Palmyra reinforced epoxy composite with different stacking sequences. It was displayed that the Izod NI strength of the composite increased almost linearly with increased fiber content up to 40 % for all stacking sequences. Karthick et al. [144] also studied the impact strength of banana fiber hybrid composite. The effect of banana fiber length (5–15 mm) and banana fiber loading (10–20 %) on the impact strength of banana/glass fiber epoxy composite. The result showed that the impact strength of the composite increases with increasing both the fiber length and fiber loadings. The maximum impact strength of the hybrid composite was obtained at 15 mm fiber length and 20 % fiber loadings. Ramesh et al. [145] analyzed the impact strength of hybrid areca/kenaf fiber reinforced epoxy composite. The result showed that 17.5 % areca with 17.5 % kenaf fiber hybrid composite revealed lower impact strength compared to 35 % of areca fiber and 35 % of kenaf fiber epoxy composite. Waheedullah et al. [146] characterized the impact strength of hybrid kenaf (K)/date palm fiber (DPF) reinforced with a constant 50 % epoxy composite. The impact strength of an equal portion of DPF/kenaf fiber reinforced epoxy composite revealed higher impact strength compared to 3K7DPF and 3DPF7K hybrid composite. The impact strength of hybrid natural fiber with synthetic fiber reinforced epoxy composite has been studied by few researchers. Balaji et al. [147] studied the impact strength of hybrid Nano silica/coir fiber reinforced with epoxy composite. The impact strength of the composite increased with increasing the coir fiber loading from 10 to 20 %. In another study, Ragunath et al. [148] evaluated the impact strength of hybrid sisal/glass fiber reinforced epoxy composite manufactured by heat compression molding techniques. The Izod NI strength of the hybrid composite improved with increasing sisal fiber content from 10 to 20 %. However, the NI strength decreased with increasing glass fiber content from 10 to 20 %. This shows that natural fiber resists more impact energy than synthetic fiber for sisal/glass-reinforced epoxy composite. In addition, the author compared the NI strength of 40 % sisal fiber reinforced epoxy composite with 40 % glass fiber reinforced composite. Surprisingly 40 % sisal-reinforced epoxy composite showed maximum NI strength compared to glass fiber epoxy composite. Vijaya et al. [149] investigated the impact strength of hybrid jute/E-glass fiber reinforced epoxy composite. The impact strength of the hybrid composite increased with increasing jute fiber content from (10–30 %) for all (0°, 15°, 30°^,^ and 45°) fiber orientations. The maximum impact strength is obtained at 0°-fiber orientation and 30 % jute fiber reinforced epoxy composite. Lokesh et al. [150] studied the impact strength of hybrid Ramie/Kevlar (6R+6K), Ramie/Glass (6R+6G) and Ramie/Kevlar/Glass (6R+4K+2G) fiber epoxy composite. The result showed that all hybrid composite revealed highest result compared to pure ramie fiber epoxy composite. On the other hand, the addition of Kevlar fiber showed the highest impact strength compared to 6R+6G composite. Moreover, the addition of both Kevlar and glass fiber exhibited the highest impact strength compared to all other hybrid composites.

## 5 Comparative study of the impact performance of natural fiber reinforced epoxy based composite

**Fig. 14:**
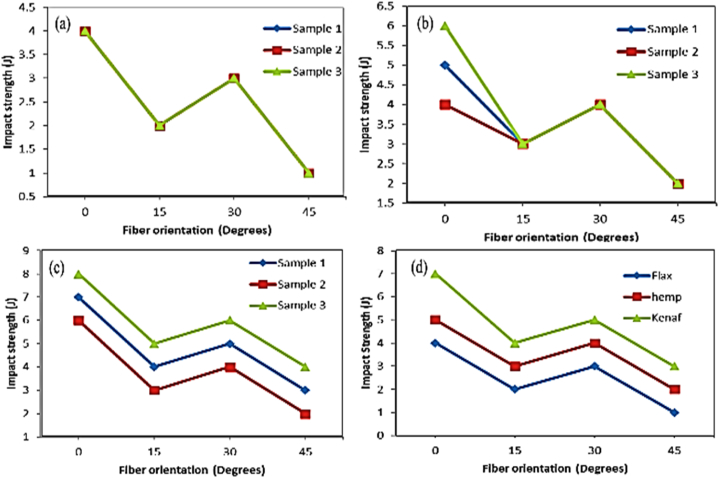
The impact strength of (a) flax fiber/epoxy, (b) hemp fiber/epoxy, (c) kenaf fiber/epoxy, and (d) all three natural fiber/epoxy composite. Reproduced with permission from Creative Commons CC BY license [122].

Comparative analysis of the impact performance of natural fiber epoxy-based composite is very essential to implement it in any applications where the impact energy is critical. The impact strength of natural fiber epoxy composite not only depends on fiber content, manufacturing techniques and chemical treatment of fiber but also on fiber type. Therefore, the impact strength of different natural fiber processing with the same manufacturing techniques and constant epoxy matrix provide different strengths. The differences in impact strength observed among natural fiber epoxy composites can be attributed to the inherent properties of the individual natural fibers, such as their tensile strength, aspect ratio, and the quality of the fiber-matrix interface. The manufacturing processes, including fiber orientation, fiber distribution, and the effectiveness of fiber impregnation, can also contribute to the variations in impact performance [94]. Researchers have shown comparative studies to identify the impact strength of pure epoxy resin against that of natural fiber-reinforced epoxy composites. Additionally, they have compared the impact performance of different types of natural fiber-reinforced epoxy composites. Julian et al. [151] performed the impact strength of fique fiber-reinforced epoxy composite and net epoxy material. The result showed that fique fiber significantly improved the Charpy NI strength of the composite compared to net epoxy material. As the author explained, fique fiber enhances the energy-absorbing capacity of the composite and improves the fragile behavior of epoxy material. Maleque et al. [152] analyzed the impact strength of pseudo-banana reinforced epoxy composite compared with net epoxy resin. It was shown that the impact strength of banana fiber-reinforced epoxy composite is approximately 40 % greater than net epoxy resin. Yang et al. [11] compared the impact strength of B.mori silk fiber/epoxy with A. pernyi silk fiber/epoxy composites. The impact strength of A. pernyi silk fiber epoxy composite revealed higher impact strength than B.mori silk fiber epoxy composite at 30 and 60 % fiber loadings. Suresh et al. [153] investigated the comparative study of NF (sisal and kenaf) reinforced epoxy composite. The author confirmed that kenaf fiber-reinforced epoxy composite has higher impact strength than sisal-reinforced epoxy composite at the same fiber ladings and processing techniques. This indicates that kenaf fiber is more compatible with epoxy composite and resists more impact energy than sisal fiber in the same manufacturing process as well as a testing method. Similarly, kenaf/epoxy composite revealed higher impact strength than sisal/epoxy composite at constant fiber loadings reported by Venkatesh et al. [123]. Osoka et al. [154] studied the impact strength of empty plantain bunch fiber, empty palm bunch fiber, and rattan palm fiber reinforced with epoxy composite. The result showed that rattan palm fiber is greater than empty palm fiber and empty plantain fiber reinforced epoxy composite. Empty palm fiber-reinforced epoxy composite has higher impact strength compared to empty plantain fiber-reinforced epoxy composite. Mrinal Kanti et al. [155] analyzed the impact strength of jute, coconut coir, and human hair-reinforced epoxy composite using the Charpy and Izod impact test. It was shown that jute epoxy composite revealed higher impact strength than coconut coir and human hair-reinforced epoxy composite. In contrast, coconut coir fiber reinforced epoxy composite exhibited the lowest impact strength when compared to human hair and jute fiber reinforced epoxy composite. In addition, the Charpy impact test of the composite showed higher results than the Izod impact test in all fiber composites. In a later study, Mrinal Kanti and Prashant. Shukla [156] conducted both Izod and Charpy impact strength of bagasse/epoxy and banana/epoxy composite. The result showed that bagasse/epoxy composite has higher impact strength compared to banana/epoxy composite. M.A. Islam et al. [157] studied the impact strength of 5 % sawdust fiber/epoxy and 5 % jute fiber/epoxy composite. The study found that jute fiber reinforced epoxy composites exhibited greater impact strength compared to composites made with sawdust/epoxy composite. This superior performance of jute fiber is attributed to its smaller diameter, which allows for better bonding with the epoxy matrix, resulting in a stronger and more impact-resistant composite. Hema Aditya et al. [158] also characterized the comparative study of natural fibers such as date palm, sisal, and pineapple-reinforced epoxy composite. The result displayed that date palm fiber reinforced epoxy composite revealed the highest NI strength compared to sisal and pineapple-reinforced epoxy composite. On the other hand, pineapple with sisal hybrid reinforced epoxy composite showed higher NI strength than sisal fiber and lower NI strength compared to pineapple-reinforced epoxy composite. Similarly, S. Krishna Mohan et al. [137] evaluated the impact strength of pure sisal/epoxy, bamboo/epoxy and sisal/bamboo hybrid reinforced epoxy composite. It can be observed that sisal/epoxy composite has higher impact energy compared to bamboo/epoxy composite. The impact strength of 12.5 % bamboo/7.5 % sisal hybrid epoxy composite showed an increment of 13 % and 31 % compared to pure bamboo and sisal-reinforced epoxy composite respectively. Narendiranath et al. [159] studied the impact strength of angora, kenaf, and ramie fiber-reinforced epoxy composite. It can be observed that kenaf fiber has the highest impact strength compared to other fibers whereas; ramie fiber has the lowest impact strength than other fiber-reinforced epoxy composite. Singh et al. [160] studied the impact strength of different natural fiber (sisal, hemp, jute, banana and abaca) fiber reinforced epoxy composites. Compared to other natural fiber epoxy composites, hemp epoxy composite revealed higher impact strength at constant fiber loadings of 40 %. Jute fiber epoxy composite exhibited better impact strength compared to sisal, banana and abaca fiber reinforced epoxy composite next to hemp fiber. The lowest impact strength obtained in abaca fiber reinforced epoxy composite compared to other natural fiber epoxy composite. P. V. Reddy et al. [161] evaluated the impact strength of Prosopis Juliflora (PJ), Abutilon Indicum (AI) and Tapsi (T) fiber reinforced epoxy composite. The impact strength of PJ/epoxy composite revealed the highest impact strength contrasted to other fiber epoxy composites up to 20 % fiber loadings. Whereas, at 25 % fiber loading AI/epoxy composite showed the highest impact strength compared to other T/epoxy and PJ/epoxy composites. V. Chaudhary et al. [162] studied the impact strength of flax, jute and hemp-reinforced epoxy composite at a constant fiber loading of 20 %. The impact strength of all fiber composite showed a significant improvement compared to pure epoxy material. On the other hand, jute/epoxy composite revealed higher impact strength compared to hemp and flax/epoxy composite. Whereas, flax/epoxy composite showed the lowest impact strength compared to other composites. Moreover, the impact strength of hybrid J/H/F/epoxy is higher impact strength than J/H/epoxy and F/H/epoxy composite. Fiber pull out, brittle nature of epoxy matrix, fiber fracture and fiber-matrix interface de-bonding are the main reasons for failure of developed composite subjected to the impact test. Rapeta Sundara et al. [122] analyzed the impact strength of hemp, flax and kenaf fiber reinforced epoxy composite at different fiber orientations (0°, 15°, 30° and 45°) as shown in Fig. 14(a–d). Similar to another study, kenaf fiber exhibited higher impact strength compared to hemp and flax fiber reinforced epoxy composite in all fiber orientations. Sarikaya et al. [163] studied the impact strength of palm/epoxy, birch/epoxy and eucalyptus/epoxy composite at a constant of 35 % fiber loading. In contrast to birch and eucalyptus/epoxy composite, palm/epoxy composite exhibited maximum impact strength. Birch/epoxy composite revealed the lowest impact strength due to its brittleness nature compared to other fibers. Nelson Raja et al. [164] evaluated the impact strength of hybrid Hair/Raavi, Hair/Neem and Neem/Raavi reinforced epoxy composite. The impact strength of Hair/Neem reinforced epoxy composite has the highest impact strength compared to Hair/Raavi and Neem/Raavi epoxy composite. The addition of hair fiber significantly improved the impact strength of the composite. Sathish et al. [165] evaluated the impact strength of different hybrid natural fiber reinforced epoxy composites at 40 % of fiber loading. It can be observed that the impact strength of the kenaf/flax/epoxy composite exhibits maximum strength compared to sisal/pineapple/epoxy, Kenaf/pineapple/epoxy, sisal/kenaf/epoxy and flax/pineapple/epoxy hybrid composites. The addition of kenaf and flax revealed maximum impact strength compared to sisal/pineapple/epoxy composite.

In summary, the addition of natural fibers significantly improves the impact resistance and reduces the brittleness of neat epoxy resin. The impact strength of different natural fiber reinforced epoxy composites with the same manufacturing techniques, fiber content, and other parameters revealed varying impact strengths. These differences are attributed to the intrinsic strength of each fiber, the compatibility between the fiber and the matrix, and the optimal fiber-to-matrix ratio. From the literature, bast fiber reinforced epoxy composite generally exhibits better impact strength than other fiber types. Among bast fibers, kenaf fiber gives higher impact strength than jute, hemp, flax and other fibers. Whereas, other natural fibers like banana, abaca, and bamboo fiber showed the lowest impact strength compared to bast fibers. Table 4 shows the impact strength of both hybrid natural fiber epoxy composites and the comparative impact strength of different natural fiber epoxy composites.

**Table 4:** Comparative impact strength of different and hybrid natural fiber epoxy based composite.

Natural fiber epoxy Composites	Fabrication techiniques	Impact strength (kJ/m^2^)	Impact strength (J/m)	Impact strength (J)	Ref.
30 % Jute/epoxy	Hand layup	_	180	_	[134]
18 % Jute+12 % Curaua/epoxy		_	380	_	
18 % Jute+12 % Sisal/epoxy		_	560	_	
50 % Jute + banana/epoxy	≫	13.44	_	_	[135]
37.5 % Jute+12.5 % banana/epoxy		15.81	_	_	
25 % Jute+25 % banana/epoxy		18.23	_	_	
12.5 % Jute+37.5 % banana/epoxy		17.89	_	_	
Jute+50 % banana/epoxy		16.92	_	_	
10 % Bamboo + sisal fiber/epox	Compression molding	_	_	3.2	[137]
10 % Sisal + bamboo fiber/epoxy		_	_	3.9	
12.5 % Bamboo+7.5 % sisal fiber/epoxy		_	_	4.4	
25 % Ramie+25 % banyan fiber/epoxy		_	_	12.12^t^ 11.14^u^	
20 % Ramie+30 % banyan fiber/epoxy	Hand layup	_	_	11.18^t^ 10.12^u^	
10 % Ramie+40 % banyan fiber/epoxy		_	_	9.04^t^ 7.04^u^	
36 % Coir fiber/epoxy	≫	2.40	_	_	[143]
20 % Coir + 16 % jute fiber/epoxy		2.72	_	_	
20 % Coir + 16 % flax fiber/epoxy		2.77	_	_	
20 % Coir + 16 % cotton fiber/epoxy		2.25	_	_	
20 % Coir + 16 % human hair fiber/epoxy		2.14	_	_	
20 % Coir + 16 % sisal fiber/epoxy		2.65	_	_	
20 % Coir + 16 % kenaf fiber/epoxy		2.85	_	_	
20 % Coir + 16 % calotrropis fiber/epoxy		2.30	_	_	
40 % Bagasse+10 % ricehusk fiber/epoxy	≫	0.22	_	_	[141]
35 % Bagasse+15 % ricehusk fiber/epoxy		0.29	_	_	
20 % Bagasse+30 % ricehusk fiber/epoxy		0.23	_	_	

Natural fiber epoxy Composites	Fabrication techiniques	Impact strength (kJ/m^2^)	Impact strength (J/m)	Impact strength (J)	Ref.
Oil palm bunch fiber/epoxy	Hand layup	1981	_	_	[154]
Plantain bunch fiber/epoxy		2020	_	_	
Rattan palm fiber/epoxy		2049.7	_	_	
Cocunet coir fiber/epoxy	≫	_	_	0.5	[155]
Jute fiber/epoxy		_	_	3	
Humain fiber/epoxy		_	_	2	
Sisal fiber/epoxy		_	65.63	_	[158]
Date palm fiber/epoxy	≫	_	106.3	_	
Pineapple fiber/epoxy		_	187.5	_	
Sisal/pineapple fiber/epoxy		_	90.63	_	
15 % Hair +15 % raavi fiber/epoxy		_	_	0.46	[164]
15 % Hair +15 % raavi fiber/epoxy	≫	_	_	0.68	
15 % Hair +15 % raavi/epoxy		_	_	0.45	
Kenaf fiber/epoxy	≫	21.5	_	1.82	[159]
Angora fiber/epoxy		19.7	_	1.64	
Ramie fiber/epoxy		17.5	_	1.55	
Sisal fiber/epoxy		100	_	_	[161]
Jute fiber/epoxy		76	_	_	
Banana fiber/epoxy	≫	86	_	_	
Hemp fiber/epoxy		86	_	_	
Abeca fiber/epoxy		86	_	_	
Jute fiber/epoxy	≫	7.68	_	_	[162]
Hemp fiber/epoxy		5.18	_	_	
Flax fiber/epoxy		4.71	_	_	
25 % (Jute + hemp) fiber/epoxy		6.93	_	_	
25 % (Hemp + flax) fiber/epoxy		4.18	_	_	
25 % (Jute + hemp + flax) fiber/epoxy		10.19	_	_	

**Note;** treated (t), untreated (u) and reference (Ref.).

## 6 Conclusion and future trends

In conclusion, this review has provided valuable insights into the impact strength of natural fiber epoxy composites, shedding light on the key factors influencing their performance and potential for various industrial applications. The review highlighted the effect of fiber content, fiber length, stacking sequence and their inherent mechanical properties in determining the impact resistance of natural fiber epoxy-based composite. Different natural fibers such as jute, hemp, flax, sisal, coir, etc have shown promise in enhancing impact strength due to their unique characteristics, including high strength and stiffness. Among polymer matrix materials available, epoxy matrix has high impact strength, low density, high resistance to temperature, and low shrinkage properties. Due to this several researchers have developed a composite material from natural fiber and epoxy as a matrix. The impact strength of natural fiber epoxy-based composites reported by different scholars was discussed in this review work. Due to the nature of fiber type, different manufacturing techniques, test conditions, fiber matrix interactions and other conditions concluding and discussing the impact strength based on fiber content is certainly challenging. From the literature, it can be concluded that natural fiber significantly improves the impact strength of epoxy-based composites regardless of other parameters. The impact strength of natural fiber epoxy-based composite increased with increasing fiber content up to a certain optimum weight in many research studies. This is because natural fiber blocks the formation of cracks in the matrix creates a wide contact area in the composite and has high energy-absorbing capacities. The impact energy of natural fiber epoxy-based composites is not only affected by fiber content but also affected by chemical treatments. Regardless of concentration and soaking time, chemical treatment increases the impact strength of the composite by enhancing the fiber/matrix interfacial bondings. The better fiber/matrix bonding the higher the impact resistance of the composite. In addition, the effect of fiber length on the impact strength of natural fiber epoxy-based composite was discussed. As reported by many research works, epoxy-based composite's impact strength increases with increasing fiber length. This is due to the formation of fiber pullout, fiber/matrix debonding, fiber breakage and formation of localized stress for short fiber epoxy-based composite.

Despite the numerous benefits of natural fiber on the impact strength of epoxy-based composites, certain challenges and limitations were identified. Fiber variability, moisture absorption, and limited fiber length were recognized as factors that can affect the impact strength. Moreover, the poor fiber/matrix bondings, hydrophilic nature of the fiber, the addition of filler materials, fiber treatments, curing times and poor manufacturing methods are factors affecting the impact strength of the composite. Therefore, in the future, researchers will address these challenges through careful fiber selection, fiber treatment techniques, good manufacturing methods and appropriate hybridization approaches can help to overcome these limitations. Additionally, the incorporation of nanomaterial reinforcements has shown promise in enhancing impact resistance by improving matrix-fiber interactions and increasing stiffness.

In summary, this review has provided a comprehensive understanding of the impact strength of natural fiber epoxy-based composites. By considering the interplay between natural fibers, matrix properties, processing techniques, and composite design, the review has identified strategies to enhance impact strength and promote the applicability of these composites in diverse industries by improving the fiber's hydrophilic nature. Continued research and development efforts in this field will further contribute to the advancement of sustainable materials with improved impact resistance, paving the way for a greener and more resilient future.

The author(s) received no financial support for this article's research, authorship, and/or publication.

## CRediT authorship contribution statement

**Abdu Mohammed Seid:** Writing – review & editing, Writing – original draft, Resources, Conceptualization. **Solomon Alemneh Adimass:** Writing – review & editing, Visualization, Formal analysis.

## Data availability statement

Data will be provided on request.

## Declaration of competing interest

The authors declare the following financial interests/personal relationships which may be considered as potential competing interests: Abdu mohammed reports was provided by Wollo University. Abdu Mohammed reports a relationship with Wollo University that includes: employment. Abdu Mohammed has patent pending to Abdu mohammed. no other additional relationships. Both corresponding author and co-author working at Wollo university If there are other authors, they declare that they have no known competing financial interests or personal relationships that could have appeared to influence the work reported in this paper.
